# Rapid Macrosatellite Evolution Promotes X-Linked Hybrid Male Sterility in a Feline Interspecies Cross

**DOI:** 10.1093/molbev/msab274

**Published:** 2021-09-14

**Authors:** Kevin R Bredemeyer, Christopher M Seabury, Mark J Stickney, John R McCarrey, Bridgett M vonHoldt, William J Murphy

**Affiliations:** 1 Veterinary Integrative Biosciences, Texas A&M University, College Station, TX, USA; 2 Interdisciplinary Program in Genetics and Genomics, Texas A&M University, College Station, TX, USA; 3 Veterinary Pathobiology, Texas A&M University, College Station, TX, USA; 4 Veterinary Medical Teaching Hospital, Texas A&M University, College Station, TX, USA; 5 Department of Biology, University of Texas at San Antonio, San Antonio, TX, USA; 6 Ecology and Evolutionary Biology, Princeton University, Princeton, NJ, USA

**Keywords:** interspecies hybrid, gene mapping, genome assembly, meiosis, macrosatellite, X chromosome

## Abstract

The sterility or inviability of hybrid offspring produced from an interspecific mating result from incompatibilities between parental genotypes that are thought to result from divergence of loci involved in epistatic interactions. However, attributes contributing to the rapid evolution of these regions also complicates their assembly, thus discovery of candidate hybrid sterility loci is difficult and has been restricted to a small number of model systems. Here we reported rapid interspecific divergence at the *DXZ4* macrosatellite locus in an interspecific cross between two closely related mammalian species: the domestic cat (*Felis silvestris catus*) and the Jungle cat (*Felis chaus*). *DXZ4* is an interesting candidate due to its structural complexity, copy number variability, and described role in the critical yet complex biological process of X-chromosome inactivation. However, the full structure of *DXZ4* was absent or incomplete in nearly every available mammalian genome assembly given its repetitive complexity. We compared highly continuous genomes for three cat species, each containing a complete *DXZ4* locus, and discovered that the felid *DXZ4* locus differs substantially from the human ortholog, and that it varies in copy number between cat species. Additionally, we reported expression, methylation, and structural conformation profiles of *DXZ4* and the X chromosome during stages of spermatogenesis that have been previously associated with hybrid male sterility. Collectively, these findings suggest a new role for *DXZ4* in male meiosis and a mechanism for feline interspecific incompatibility through rapid satellite divergence.

## Introduction

Hybrids between mammalian species are often infertile or inviable as a result of genetic incompatibilities between parental haplotypes, as described by the “two rules of speciation” (Coyne and Orr 1997; Coyne 2018). The first, Haldane’s rule, is the long-standing observation of preferential sterility or inviability of the heterogametic sex resulting from an interspecific mating (Haldane 1922). The second rule is the large X-effect (Coyne 1992), which is the observation that the X chromosome is enriched for hybrid sterility factors and plays an increased role in postzygotic isolation relative to autosomes (Coyne 1992; Masly and Presgraves 2007). Support for the large X-effect in mammals has come from genetic mapping studies of sterility phenotypes to the X chromosome using genome-wide association, QTL and eQTL studies (Good et al. 2008; Bhattacharyya et al. 2014; Turner et al. 2014; Turner and Harr 2014; Schwahn et al. 2018; Lustyk et al. 2019).

Gene expression comparisons between fertile and sterile hybrid mouse testes revealed that X-linked genes in sterile testes were highly upregulated across the X chromosome (Good et al. 2010). Subsequent hybrid sterility studies employing enriched germ cell populations revealed X upregulation occurred in all stages of spermatogenesis but was most pronounced in cell stages that undergo meiotic sex chromosome inactivation (MSCI) and exhibit X-chromosome downregulation during normal spermatogenesis (Namekawa et al. 2006; Campbell et al. 2013; Larson et al. 2016). MSCI normally results in silencing and partitioning of both sex chromosomes into a heterochromatic XY body during pachynema (Solari 1974; Handel 2004; Turner 2007). MSCI likely evolved in response to the extensive X−Y asynapsis, which is a consequence of the gradual increase in structural and sequence divergence of the differentiating X and Y gametologs during early stages of sex chromosome evolution (Lahn and Page 1999; Graves 2006; Liu 2019). MSCI is a specific version of a more general mechanism, termed meiotic suppression of unsynapsed chromatin (MSUC), which silences any asynaptic regions between homologous molecules (Schimenti 2005; Turner et al. 2005). Although both mechanisms occur during the pachytene stage of meiosis I, MSUC is often caused by high sequence divergence or structural variation between otherwise homologous chromosomes (Wang and Höög 2006), whereas MSCI results from the heteromorphic nature of mammalian sex chromosomes specifically (McKee and Handel 1993). The failure of the X chromosome to undergo proper conformational changes due to intrachromosomal structural variation has been hypothesized as an underlying mechanism explaining failure of MSCI, which manifests as X-chromosome upregulation during spermatogenesis of sterile hybrids (Lifschytz and Lindsley 1972; Jablonka and Lamb 1991).

Although previous studies have linked the failure of MSCI or postmeiotic sex chromosome repression (PSCR) to pachytene arrest and male sterility (Burgoyne et al. 2009; Good et al. 2010; Royo et al. 2010; Larson et al. 2016; Schwahn et al. 2018), and identified genes required for proper regulation of MSCI (Vernet et al. 2016), a genetic mechanism is currently lacking to explain how X-chromosome divergence triggers the failure of MSCI and X-linked gene upregulation in the context of hybrid sterility. Our previous studies demonstrated that two biomarkers of sterility in hybrid mice testes, X chromosome-wide upregulation and meiotic arrest during pachynema, were conserved in cat interspecific hybrids (Davis et al. 2015). This suggested sterile hybrids from divergent mammalian orders could conceivably result from a similar mechanism.

Here, we explore hybrid male sterility in an interspecific hybrid cat breed, the Chausie. Chausies are an admixed cat breed population initially derived from a small number of foundational crosses between male Jungle cats (*Felis chaus*) and female domestic cats (*Felis silvestris catus*). We identify a novel mammalian X-linked candidate hybrid sterility locus, *DXZ4*, using a combination of GWAS, ancestry-based fine mapping, and genome-wide methylation approaches. We used sorted germ cells from a fertile domestic cat to generate expression, methylation, and chromatin profiles of *DXZ4* and the X chromosome during two relevant stages of spermatogenesis. Our results indicate that *DXZ4* plays a heretofore undescribed role in normal male meiosis, and that interspecific divergence across this locus likely contributes to disruption of MSCI, manifesting as hybrid sterility.

## Results and Discussion

### Biomarkers of Chausie Hybrid Male Sterility

**Fig. 1.**
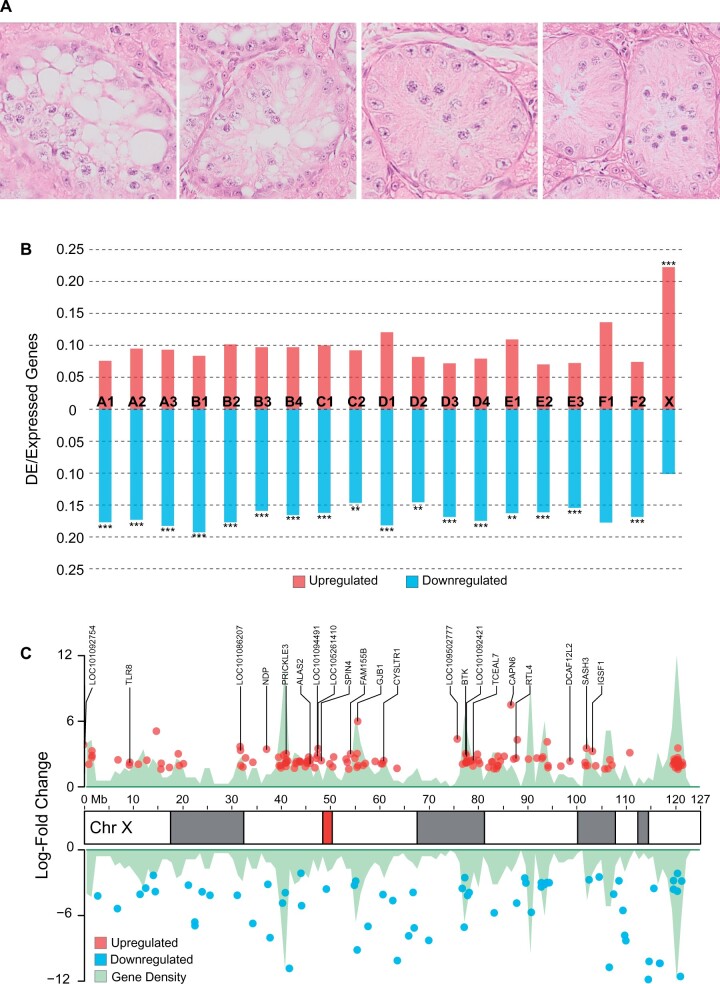
Chausie F1 males exhibit two biomarkers commonly associated with hybrid male sterility in mammals. (*A*) Histological cross-sections of seminiferous tubules from sterile testes indicate arrest of spermatogenesis during pachynema. The two left-most images show vacuolization of the seminiferous epithelium, and the two right-most images show depletion of post pachytene germ cells. (*B*) Differential expression analysis of RNA-Seq data from sterile and fertile testes shows the X chromosome in sterile testes shows a significant number of upregulated genes relative to the null hypothesis of no difference between up and downregulated genes on a per chromosome basis, with autosomes showing the opposite pattern (Chromosomes A1−F2). *P* values: *≤0.05, **≤0.01, ***≤0.001. (*C*) Distribution of genes differentially expressed on the X chromosome in sterile testes. Labeled genes were those upregulated in sterile testes and lacking expression in fertile testes. In the X chromosome ideogram, the centromere is red and heterochromatic G-bands are gray.

We analyzed histology and RNA-Seq data from fertile and sterile testes from Chausies of fourth and fifth backcross generations containing similar pedigree-based estimated percentages (13−14%) of Jungle cat ancestry. Histological analysis of seminiferous tubule cross-sections from sterile Chausies revealed vacuolization, depletion of postpachynema germ cells, and meiotic arrest at the pachytene spermatocyte stage, similar to observations from testes of sterile males in other hybrid cat breeds (fig. 1*A*) (Davis et al. 2015) and many other mammalian species (Moore et al. 1999; Thomsen et al. 2011; Bhattacharyya et al. 2013; Ishishita et al. 2015).

Transcriptional profiling of seminiferous tubule RNA isolates from sterile Chausies revealed upregulation relative to fertile males in 22% of annotated protein coding genes along the X chromosome, a significant enrichment relative to autosomes (Fisher’s exact test, *P* value = 1.5e−21). Expression of X-linked genes increased by a fairly uniform 2.45 log-fold average across the length of the chromosome (fig. 1*B*). There was no apparent clustering of differentially expressed (DE) genes along the length of the X chromosome (fig. 1*C*), consistent with a pattern of chromosome-wide misregulation as opposed to regional escape of meiotic gene silencing, concordant with previous observations from felid interspecific and rodent subspecific hybrids (Good et al. 2010; Davis et al. 2015; Larson et al. 2016). X upregulated genes were not significantly enriched for any biological processes. Twenty-two genes that were overexpressed in sterile Chausies showed zero expression in normal, fertile testes from domestic cats or Chausies (fig. 1*C*). These genes exhibited an average log-fold increase of 3.28, significantly larger than the fold change of genes normally expressed in fertile testes (Student’s two-tailed *t*-test, *P* value = 0.002). These results suggest the observed chromosome-wide misregulation in sterile Chausies was likely due to defective MSCI, and not an artifact of tissue composition bias (Good et al. 2010). Interestingly, we also observed significant numbers of genes downregulated across almost all autosomes in these testis samples (chi-squared test, *P* value = 2.2e−16) (fig. 1*B*;supplementary fig. 1, Supplementary Material online), a feature not previously observed in other felid interspecific hybrids (Davis et al. 2015), despite sharing a similar meiotic arrest phenotype. Downregulated genes genome-wide were enriched for biological processes involved in meiotic division and spermatogenesis (supplementary table 1, Supplementary Material online), suggesting the apparent downregulation of many of these genes likely stems from an absence of postpachytene germ cells.

### GWAS and Fine Mapping of Hybrid Sterility Locus DXZ4

**Fig. 2.**
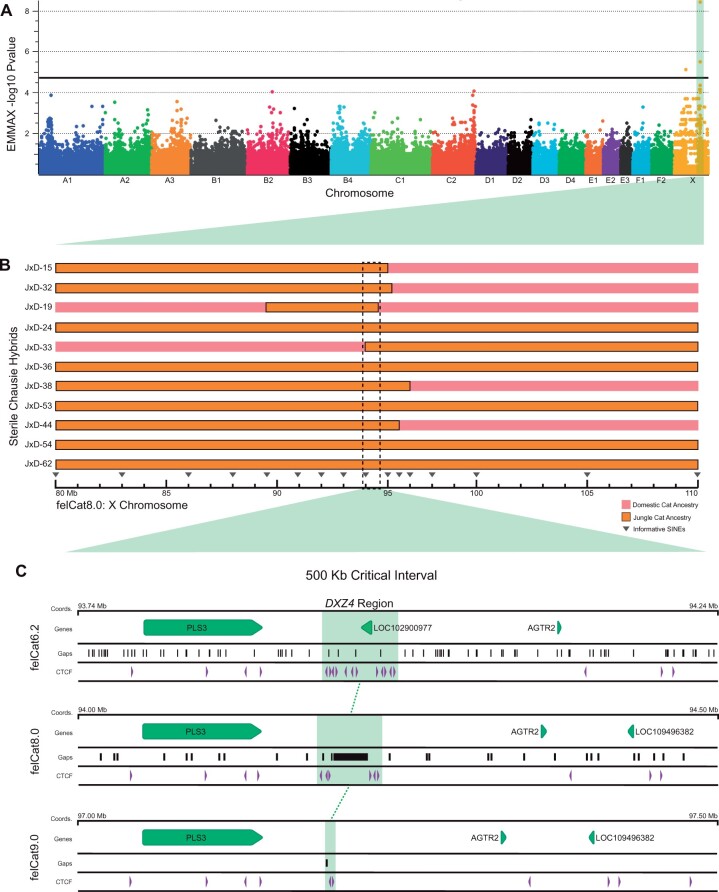
Identification of hybrid sterility locus *DXZ4* in a cohort of male Chausie backcross hybrids. (*A*) Hybrid sterility GWAS in a cohort of sterile and fertile Chausie hybrids identifies a significant SNP peak across the 94 Mb region of the felCat8 X chromosome exceeding the Wellcome Trust recommendations for genome-wide significance (*P*_uncorrected_<5 × 10^−5^; −log_10_*P* = 4.30). (*B*) Fine-mapping studies in Chausie backcross hybrids. A 500-kb critical interval was identified by associating regions of shared Jungle cat ancestry with the sterile phenotype (only the most informative hybrids are shown). Jungle cat ancestry for this region was observed in 14/16 sterile Chausie hybrids, whereas domestic ancestry was observed in all fertile hybrids (*n* = 23). (*C*) Macrosatellite *DXZ4* identified as candidate hybrid sterility locus with CTCF-binding sites flanking assembly gaps and conserved position downstream of *PLS3*.

To identify genetic variation associated with the hybrid male sterility phenotype, we performed a genome-wide association study (GWAS) by genotyping a cohort of 39 backcross hybrid males (phenotyped for sterility as described in Materials and Methods) on the Illumina 63K feline array. A single cluster of significant SNPs was identified on the X chromosome at ∼94 Mb in the felCat8.0 reference assembly (fig. 2*A*). We performed fine-mapping of the candidate SNP region by genotyping 27 ancestry-informative short-interspersed element (SINE) insertions identified from whole genome sequence alignments that distinguished the domestic cat and Jungle cat X chromosomes. This approach identified a 500-kb critical interval between 93.74 and 94.24 Mb in 94% of backcross hybrid males that possessed Jungle cat ancestry and were sterile. In contrast, 100% of male Chausies that inherited a domestic cat haplotype within this region were fertile (fig. 2*B*).

Three annotated functional loci reside within the critical interval region, two protein coding genes (*PLS3 and AGTR2*) and a large macrosatellite repeat (MSR) array (*DXZ4*) that has a well-documented role in primate and rodent female X-chromosome inactivation (XCI) (Deng et al. 2015; Darrow et al. 2016). The two protein coding genes within this interval were excluded from consideration based on the following functional genomic data. The first gene, *PLS3*, has a broad tissue expression profile with relatively low expression in testis reported in the NCBI genome browser and has no described role in spermatogenesis (supplementary fig. 2, Supplementary Material online). The second gene, *AGTR2*, is not expressed in the feline testis. Therefore, we explored *DXZ4* as the candidate locus.

*DXZ4* is a macrosatellite, a subcategory of variable number tandem repeat sequences distinguished by its large, multikilobase repeat unit (Giacalone et al. 1992; Dumbovic et al. 2017). *DXZ4* plays a well described role in structural aspects of female XCI that occurs in female somatic cells and results in the inactivation of a single X chromosome to balance gene dosage with hemizygous males (Lyon 1961; Brockdorff and Turner 2015; Galupa and Heard 2018; Bansal et al. 2019). Given this previously described role in XCI, we were intrigued by the strong genotypic association based on *DXZ4* ancestry in males: 94% of sterile hybrids possessed a Jungle cat *DXZ4* allele within a mostly domestic cat genetic background, whereas all fertile hybrids possessed a domestic cat *DXZ4* allele. Prior to this observation, there was no evidence that *DXZ4* had a functional role in male meiotic silencing of sex chromosomes despite numerous parallels between X chromosome states resulting from male (i.e., MSCI) and female (i.e., XCI) silencing mechanisms. The final heterochromatic state is similar between the two silencing processes despite differences in certain histone modifications and differences in DNA methylation (McCarrey et al. 1992; Armstrong et al. 1997; Hoyer-Fender 2003; Moretti et al. 2016), as is the segregation of the inactive X from autosomes through association with the nucleolus (Kierszenbaum and Tres 1974; Knibiehler et al. 1981). During XCI, the shift in localization is governed by *DXZ4* and lncRNA from the associated locus *FIRRE*, which anchor the inactive X (Xi) to the nucleolar periphery (Deng et al. 2015; Yang et al. 2015; Fang et al. 2020). This localization results in physical interaction between *DXZ4* and additional X-linked MSR loci, *FIRRE and ICCE*, which form large DNA superloops visible in Hi-C interaction maps (Darrow et al. 2016; Jégu et al. 2017). In XCI, nucleolar association is thought to govern the Xi epigenetic state (Zhang et al. 2007).

Although localization of the X chromosome to the nucleolar periphery occurs during MSCI, its significance, as well as the role *DXZ4* might play in maintaining this state, is unknown. In addition to epigenetic and spatial similarities, distinct repertoires of miRNAs escape X chromosome silencing in each instance, suggesting a common role for posttranscriptional regulation and the potential for shared means of gene escape between silencing mechanisms (Yan and McCarrey 2009; Sosa et al. 2015). To evaluate *DXZ4* expression and methylation patterns during spermatogenesis, we profiled three different functional properties of *DXZ4* in whole testes and sorted germ cells from sexually mature domestic cats: 1) transcription using RNA-Seq, 2) methylation using reduced representation bisulfite sequencing (RRBS), and 3) long-range chromatin interactions of the silenced X chromosome using Hi-C.

### DXZ4 Assembly and Structural Assessment

Because of its complex structure and polymorphic nature, *DXZ4* is incomplete or altogether missing in even the highest quality genome assemblies (fig. 2*C*) and has been poorly studied in most placental mammals. We first generated contiguous assemblies across the *DXZ4* macrosatellite in both parent species of the Chausie cat hybrids to enable a comparative analysis of locus structure and copy number and facilitate examination of X-chromosome expression and methylation in the male germ line. *DXZ4* was annotated adjacent to an assembly gap in all previous iterations of the domestic cat reference genome assembly (Montague et al. 2014; Li, Hillier, et al. 2016; Buckley et al. 2020) (fig. 2*C*). Comparisons between the different genome assemblies revealed substantial variation in the proportion of *DXZ4* sequence successfully incorporated into the X chromosome, likely due to the different sequence chemistries employed and their variable average read lengths (supplementary fig. 3, Supplementary Material online). Fortuitously, the *DXZ4* locus was fully assembled in our recently published single haplotype genome assemblies for the domestic cat and Asian leopard cat (Bredemeyer et al. 2021) (supplementary fig. 4, Supplementary Material online). We additionally sequenced and assembled the genome of a male Jungle cat (where the X chromosome is effectively haploid) from long PacBio sequence reads (supplementary discussion and supplementary table 2, Supplementary Material online). This Jungle cat assembly (FelChav1.0) was highly contiguous and contained within 106 contigs, totaling 2.43 Gb, with a contig N50 = 91 Mb and scaffold N50 = 148.6 Mb (table 1).

**Table 1.** FelChav1.0 Assembly Statistics.

Species	Jungle Cat (2*n* = 38)
Read count	7,594,421
Base count (bp)	122,681,343,250
Subread N50 (bp)	25,928
Contig assembly	
Total contigs	106
Largest contig (bp)	205,710,267
Ungapped assembly length (bp)	2,428,281,414
N50 (bp)	91,188,488
BUSCO (mammalia_odb10)	
Single-copy	8,559
Duplicated	25
Complete	8,584
Percent complete	93.04%
Fragmented	180
Missing	462
Percent present (Comp + Frag)	94.99%
Scaffold assembly stats	
Total scaffolds	52
Primary assembly length (bp)	2,428,287,114
Total gaps	61
N50 scaffold (bp)	148,552,997

**Fig. 3.**
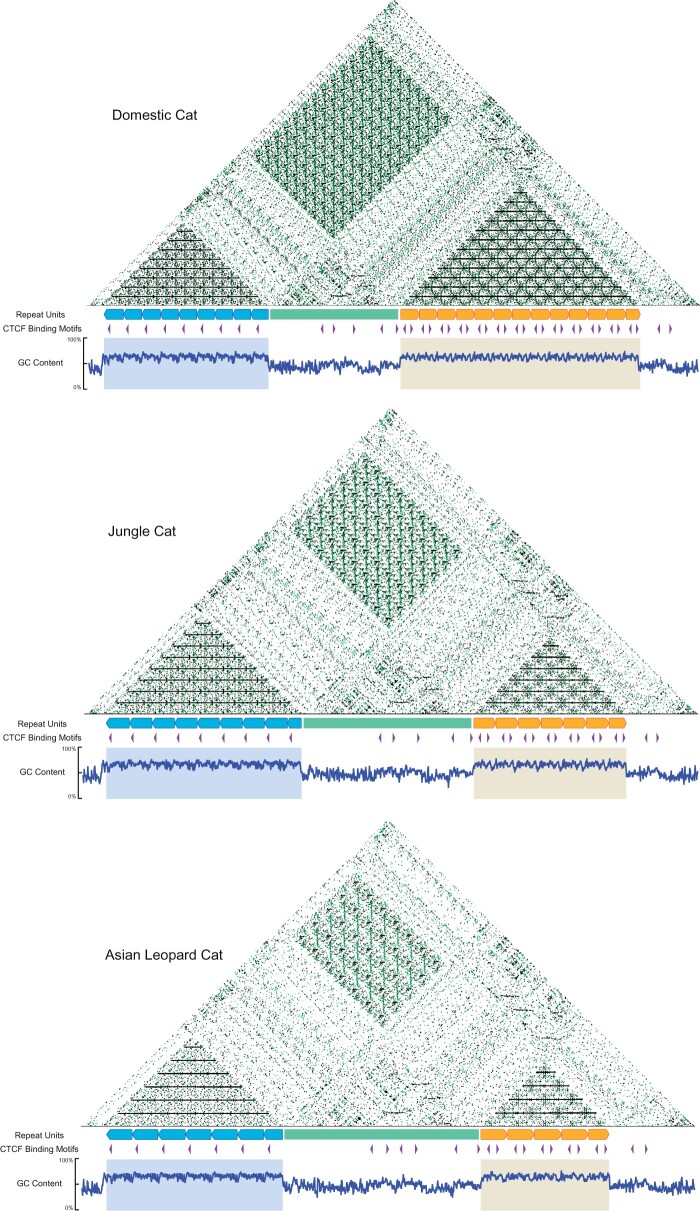
Dichotomous structure of *DXZ4* revealed in three cat species. Self-dot plots and CTCF motif annotations reveal that macrosatellite *DXZ4* is composed of two distinct tandem repeats (Repeat A = Blue, Repeat B = Orange) divided by a conserved spacer sequence (Green) in cats. Enrichment for CpG islands as suggested by high GC content across repeat units is consistent with previous observations of the locus in human and mouse. Black and green dots represent self and inverted alignments, respectively.

In all three felid assemblies, *DXZ4* was embedded within a single contig and exhibited sequence gain relative to the gapped reference felCat9, suggesting successful assembly of the locus (supplementary fig. 4, Supplementary Material online). *DXZ4* maintained its position downstream of *PLS3*, consistent with other mammalian genomes (Horakova, Calabrese, et al. 2012) (supplementary fig. 5, Supplementary Material online). In each of the single haplotype felid X chromosome assemblies, *DXZ4* is composed of a compound satellite repeat with two divergent tandem repeats, divided by a conserved spacer sequence (fig. 3). The repeat array proximal to *PLS3*, hereby referred to as Repeat A (RA), contains a single CTCF site in the reverse direction, a conserved characteristic of repeat units that comprise the monomeric human *DXZ4* repeat array (Miga et al. 2020). The more distal Repeat B (RB), on the other hand, possesses two CTCF sites facing away from one another. The intervening spacer sequence was 32.15, 34.98, and 33.97 kb long in domestic cat, Jungle cat, and Asian leopard cat assemblies, respectively. Alignments between the spacer regions revealed five conserved CTCF-binding motifs of varying directionality and approximately half the sequence divergence observed between species for RA and RB monomers (average interspecific *P* distance: Spacer = 0.015; RA and RB = 0.026) (supplementary fig. 6 and supplementary table 3, Supplementary Material online).

**Fig. 4.**
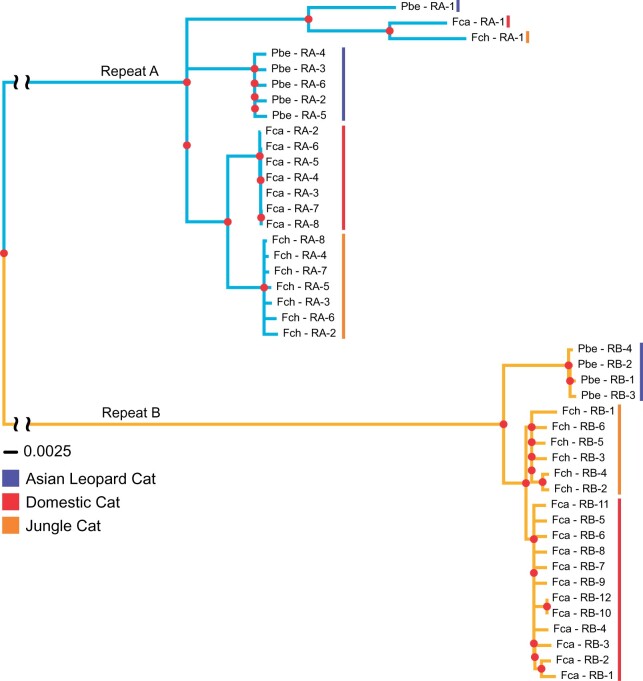
*DXZ4* repeat unit phylogenetic analysis. Neighbor joining tree generated from an alignment of felid *DXZ4* repeat units. Repeats A and B are represented from domestic (Fca), Jungle (Fch), and Asian leopard (Pbe) cats with a *DXZ4* repeat unit from the human telomere to telomere assembly (Miga et al. 2020) serving as an outgroup to root the tree (data not shown). Red dots represent a bootstrap support value ≥90.

In addition to differences in motif profiles, we observed instances of copy number variation between the tandem repeats in the three felid species. The average interspecific length of RA and RB was 4,554 and 4,607 bp, respectively, with a standard deviation (SD) of only 46 bp between all repeat units (supplementary table 4, Supplementary Material online). A maximum likelihood phylogeny of the repeat units resolved RA and RB into reciprocally monophyletic groups, with a large, between-group mean *P*-distance = 0.424. Mean within-group genetic distances were 15- to 30-fold smaller: 0.028 between RA units and 0.013 between RB units. Within RA and RB arrays, the repeat sequences grouped by species with the exception of the most proximal repeat, RA-1, which formed a divergent clade that also follows the species tree (Li et al. 2019) (fig. 4).

Despite capturing *DXZ4* within single contigs and overall structural conservation across all three cat assemblies, repeat array lengths (RA: 31 − 41 kb, RB: 22 − 59 kb, Full Array: 105 − 150 kb) far exceeded the PacBio mean read length (16 − 17 kb). We subsequently estimated the copy number with an in silico approach that utilized short-read mapping and collapsed repeat arrays (Lucotte et al. 2018) (supplementary fig. 7, Supplementary Material online). This approach was used to validate copy number for the assembly and investigate copy number variation across multiple individuals. We confirmed that copy number of RA and RB differed between all three species (supplementary table 5, Supplementary Material online). Copy number of *DXZ4* repeat arrays within domestic cats varied dramatically, especially across RA (SD = 6.81), which had twice the SD of RB (SD = 2.76). SD for domestic cat total copy number was also very high (SD = 8.45), but was expected based on the hypervariability of the locus described in human (Tremblay et al. 2011; Schaap et al. 2013). Subdivision of domestic cats based on breeding history reveals outbred domestic shorthairs display increased variation in copy number relative to defined breeds. Despite variability across individuals, the relative relationship between copy number of RA and RB remained constant within each species, with domestic cat RA copy number being less than RB on average whereas the Jungle cat and Asian leopard cat average copy number of RA exceeded that of RB.

### DXZ4 Expression in Male Germ Cells

We next asked how interspecific variation at *DXZ4*, a locus associated with a seemingly unrelated process in female somatic cells (XCI), could contribute to hybrid sterility in males. XCI requires the presence of the lncRNA *XIST*, as well as a host of long-range chromatin interacting loci (including *DXZ4*) and epigenetic modifiers (Jégu et al. 2017; Bansal et al. 2019). These loci work together to alter the structural, epigenetic, and transcriptional landscape of the inactivated X chromosome (Xi), ultimately resulting in the condensation and nucleolar association of the heterochromatic Barr body (Barr and Bertram 1949; Bourgeois et al. 1985; Dyer et al. 1989; Chadwick and Willard 2003). We hypothesized that feline *DXZ4* has a structural and/or functional role during MSCI, specifically in the formation or maintenance of the XY body formed from meiotic silencing of unsynapsed chromatin between the X and Y chromosome. This XY body is analogous to the Barr Body that forms during XCI. If true, we secondarily posited that interspecific variation at *DXZ4* might perturb XY body formation as a result of altered 3D chromatin interactions or altered gene expression that lead to the observed upregulation of X-linked genes and meiotic arrest in sterile Chausies.

Using our fully assembled *DXZ4* loci, we first investigated transcriptional activity during spermatogenesis in fertile domestic cats. In human XCI, each *DXZ4* repeat unit is capable of transcribing smallRNAs, whereas *DANT1 and DANT2* genes both transcribe multiple isoforms described as either short and array-traversing transcripts (ATTs), with the latter spanning the entire macrosatellite from flanking promotors (Figueroa et al. 2015). Although the precise function of the transcribed lncRNAs and smallRNAs are still poorly understood, they are thought to contribute to regulation of the inactive chromatin state (Pohlers et al. 2014).

**Fig. 5.**
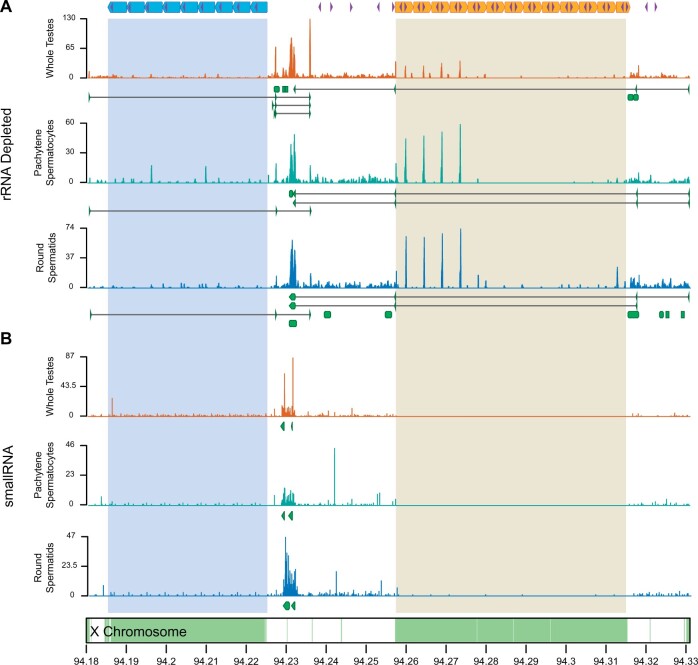
Transcriptional activity of *DXZ4* during domestic cat male meiosis. RNA-Seq data from (*A*) rRNA depleted and (*B*) smallRNA libraries generated from seminiferous tubules, sorted pachytene spermatocytes, and sorted round spermatids from a fertile domestic cat. Green regions along the X chromosome ideogram (bottom) represent CpG islands. Shaded regions across tracks represent the boundaries of the *DXZ4* repeat arrays (top). The *x*-axis corresponds to X chromosome coordinates (in Mb) in the single haplotype domestic cat genome assembly. The *y*-axis represents raw coverage scaled by maximum coverage on a per track basis. Green blocks and arrows represent exons of transcripts annotated by Cufflinks.

Analysis of rRNA-depleted and smallRNA-enriched RNA-Seq data from sorted germ cells and seminiferous tubules, revealed transcription of *DXZ4* in both pachytene spermatocytes and round spermatids, indicating that the locus escapes X silencing after MSCI and during the formation of postmeiotic sex chromatin (fig. 5). The largest peaks of transcriptional activity occurred across the spacer region adjacent to the RA array and not within individual repeat units of either array (fig. 5*A*). De novo transcript assembly identified multiple transcripts spanning the *DXZ4* locus that vary across cell types with lengths between 364 and 1,321 bp. A single 426-bp repeat-A-spanning transcript (*RAST*) similar in orientation and positioning to the human *DXZ4* array-traversing *DANT1-ATT* isoform was annotated in domestic cat whole testes, pachytene spermatocytes, and round spermatids. Annotation of the RB region also revealed a 601-bp repeat-B-spanning transcript (*RBST*) in all cell types. The orientation and positioning of *RBST* appeared orthologous to the *DANT2-ATT* isoform in humans (supplementary fig. 8, Supplementary Material online). Orthology between array spanning transcripts in cat and human was further supported by ungapped pairwise alignment identities of 46% and 47% between *RAST*/*DANT1-ATT and RBST*/*DANT2-ATT* mRNAs, respectively. Although this sequence similarity would be considered low for protein-coding sequences, these results are not unexpected for lncRNAs. LncRNAs evolve rapidly between species and exhibit signatures of conservation outside of overall sequence identity and that vary according to the functional role of the RNA within the nucleus (Kutter et al. 2012; Dhanoa et al. 2018; Ramírez-Colmenero et al. 2020). We observed many islands of consecutive conserved bases across our alignments, indicative of evolutionary constraint in DNA interacting motifs/domains or lncRNA structural conformation (Ramírez-Colmenero et al. 2020) (supplementary fig. 9, Supplementary Material online).

Additional cell type-specific transcripts were observed within and directly downstream of RB. In sorted germ cells and whole testes, we observed coverage peaks spanning ∼250 bp in each of the first four (most proximal) RB units (fig. 5*A*). Discontiguous mega BLAST analysis of the sequences underlying the peaks revealed no significant matches to the nucleotide collection database, suggesting this transcript type is unique to domestic cat. Annotation of the smallRNAs revealed two smallRNA clusters that vary only slightly in lengths and positioning between the two cell types across the spacer sequence that separates RA and RB (fig. 5*B*). SmallRNA annotations were absent from both RA and RB repeat arrays, despite visible coverage peaks of varying length across individual RA units. However, these patterns were lacking in RB units, suggesting differences in smallRNA transcription between the two arrays.

Expression across *DXZ4* was also detected in Jungle cat seminiferous tubule RNA-seq data. Similar to the domestic cat, RA- and RB-spanning transcripts were detected, with one additional RA- and two additional RB-spanning species-specific transcript variants annotated (supplementary fig. 10, Supplementary Material online). The domestic cat *RAST* and Jungle cat RA-Spanning Transcript 1 (*RAST1*) were highly similar, whereas the Jungle cat specific RA-Spanning Transcript 2 (*RAST2*) differed by a single exon in the region upstream of repeat A, suggesting divergence of exon usage between the two species (supplementary fig. 11, Supplementary Material online). We observed 96% sequence identity across alignments between conserved exons in the domestic cat *RAST* and Jungle cat *RAST1/2*. Unlike the two Jungle cat *RAST* isoforms, all RB Spanning Transcripts (*RBST1*, *RBST2*, and *RBST3*) exons were conserved between the two species and differed only in exon length, resulting in 99%, 98%, and 96% sequence identity. Despite similarities between the larger, repeat-spanning transcripts, smallRNAs were not annotated across the Jungle cat *DXZ4* region. However, we cannot rule out sample-specific bias as only a single testis was analyzed. The Jungle cat lacks annotations across the spacer region, although it appears to be depleted for smallRNA across the entire *DXZ4* locus except for RB (supplementary fig. 12, Supplementary Material online). Closer inspection of the RB-1 repeat unit in both species also revealed differences in expression peaks for the rRNA-depleted RNA libraries. In the Jungle cat, we observed large coverage peaks within the two most proximal repeat units (supplementary fig. 13, Supplementary Material online). Although similar to the ∼250-bp peaks expressed across RB 1−4 in domestic cat, peaks in the Jungle cat are larger (∼350 bp) and differ in the total number of repeats exhibiting expression, as well as the position of transcription within each repeat unit.

In summary, we observed transcriptional activity across *DXZ4* in both Jungle cat and domestic cat whole testes, as well as domestic cat sorted germ cell populations. The felid *DXZ4* locus transcribes conserved lncRNAs that are orthologous to human *DANT1 and DANT2*. We observed several clear differences in ncRNA and smallRNA expression across the RB repeat unit between the two cat species (supplementary fig. 14, Supplementary Material online). However, we cannot rule out that these apparent differences are the result of assaying expression from Jungle cat whole seminiferous tubules and not sorted germ cells. Nonetheless, our transcriptional analyses suggest that feline *DXZ4* normally escapes MSCI and plays an unknown functional role during male meiosis, possibly related to transcriptional silencing of the sex chromosomes.

### DXZ4 Methylation and Expression in Backcross Hybrids

Like other macrosatellites, *DXZ4* is subject to regulation via direct DNA methylation of enriched CpG sites. *DXZ4* was first associated with XCI when Giacalone (1992) reported that the hypomethylated state of CpG islands on the human inactive X chromosome (Xi) contrasted with the surrounding hypermethylated heterochromatin, suggesting *DXZ4* escaped silencing. Further investigation by Chadwick (2008) verified that these epigenetic differences within the Xi influenced both transcriptional activity and binding of CTCF proteins critical to normal XCI (McLaughlin and Chadwick 2011; Horakova, Moseley, et al. 2012; Bonora et al. 2018).

**Fig. 6.**
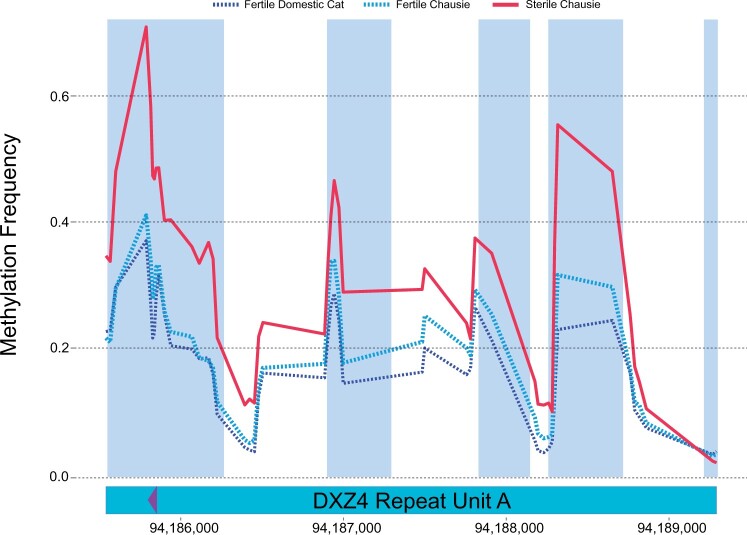
Methylation profiles across the *DXZ4* RA region in sterile and fertile hybrid testes. Sliding window of 20-cytosine averages of MF with a 10-cytosine step comparing fertile felids (*n* = 4, domestic cat = 2, Chausie = 2) and two sterile Chausies across tandem repeat A of candidate gene *DXZ4*. Regions highlighted in blue possess a window averaged *P* value of ≤0.05.

A principal component analysis (PCA) based on methylation frequency (MF) for six testes samples revealed distinct clustering of the two sterile Chausies (JXD-019, JXD-061) separate from fertile Chausies (JXD-049, JXD-080) and domestic cats (FCA-4048, FCA-4415) (supplementary fig. 15, Supplementary Material online). Methylation varied across chromosomes and fertility phenotypes (supplementary fig. 16, Supplementary Material online), with fertile felids significantly hypermethylated genome-wide relative to sterile felids (average MF: fertile = 0.220, sterile = 0.194, *t* = 2.3, df = 7.8, *P* = 0.0484). We found variable levels of methylation across the entire *DXZ4* candidate locus as well as a suggestive, but nonsignificant, trend that sterile Chausies with Jungle cat *DXZ4* ancestry (*n* = 2) were hypermethylated relative to fertile individuals with the domestic *DXZ4* haplotype (*n*, fertile Chausies = 2, domestic cat = 2) (*t* = 4.8, df = 1.5, *P* = 0.0727) (supplementary table 6, Supplementary Material online). Mean MF of the sorted pachytene spermatocytes and round spermatids revealed greater hypomethylation across the *DXZ4* region than fertile whole testis, consistent with a relaxed, open chromatin state during these stages of meiosis. Within *DXZ4*, we did observe significant hypermethylation of RA, but not RB, in sterile felids (RA: *t* = 7.1, df = 1.7, *P* = 0.0318; RB: *t* = −1.1, df = 1.1, *P* = 0.4659) (fig. 6; supplementary table 7, Supplementary Material online). Differential expression analysis between seminiferous tubules of fertile and sterile Chausies also revealed significant downregulation (log FC = 2.9) of the domestic cat *RAST* in sterile hybrids, implicating RA interspecific variation in the failure of MSCI through *DXZ4* misregulation.

In summary, we observed reversal of *DXZ4* to a hypermethylated state in sterile seminiferous tubules, associating *DXZ4* activity with the fertility status of hybrid individuals. The observed hypermethylated state of the locus in sterile males is comparable to the hypermethylated and inactivated state of human *DXZ4* on the active X chromosome (Xa) in female somatic cells. Taken together, these different lines of evidence suggest that interspecific divergence at *DXZ4* leads to transcriptional and epigenetic misregulation of *DXZ4* in the testes of sterile hybrids, and contributes to the observed biomarkers of sterility: X chromosome-wide upregulation and meiotic arrest at pachynema.

### Structural Conformation of the X Chromosome in Male Germ Cells

Previous studies revealed that the human, macaque, and mouse Xi exhibit a unique structural arrangement composed of two large super-domains forming a bipartite structure, with *DXZ4* functioning as the hinge region (Rao et al. 2014; Deng et al. 2015; Darrow et al. 2016). Knockout studies of *DXZ4* demonstrated that deletion of the locus was sufficient to disrupt the unique structural organization of the Xi in female somatic cells (Darrow et al. 2016; Giorgetti et al. 2016; Bonora et al. 2018; Bansal et al. 2019). In domestic cat, this bipartite structure is also maintained and clearly visible in Hi-C maps generated from female fibroblasts, suggesting *DXZ4* is functionally conserved in XCI of carnivores, in addition to primates and rodents (supplementary fig. 17, Supplementary Material online) (Brashear et al. 2021).

**Fig. 7.**
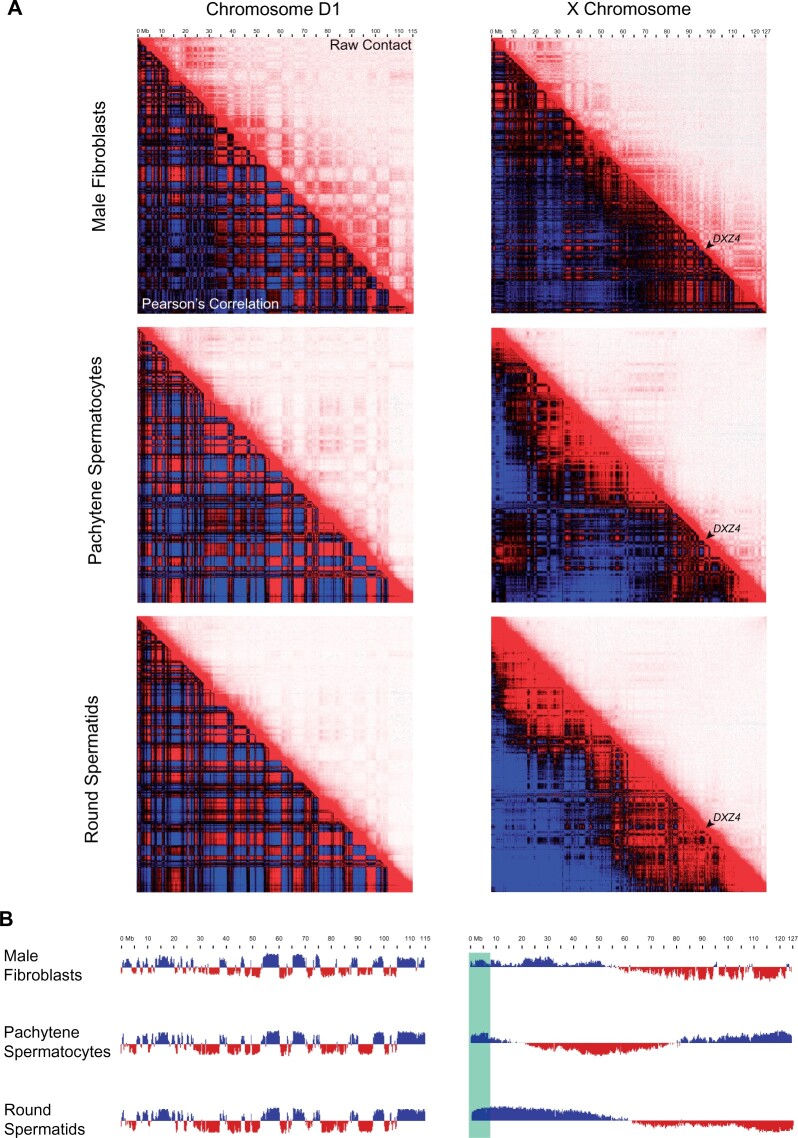
Chromatin conformation of the X chromosome in three different male domestic cat cell types. (*A*) Pearson’s correlation (bottom left)/raw contact (top right) maps of a representative autosome, Chr. D1, and the X chromosome in fibroblasts, pachytene spermatocytes, and round spermatids (Pearson’s and raw contact maps calculated at 250 kb resolution). (*B*) Eigenvector tracks revealing changes in compartmentalization during felid meiosis. The association between eigenvector directions and A/B compartments were not established using histone modification profiles for either chromosome. Blue compartments along the X chromosome are assumed to be A (active) due to escape of the pseudoautosomal region (Green) from silencing during MSCI/PSCR (Turner et al. 2001; de Vries et al. 2012).

Because of the interesting parallels between silenced X chromosomes resulting from MSCI and XCI, we sought to compare changes in chromatin conformation resulting from each process. We used previously published Hi-C data from a female F1 Bengal hybrid cell line (Bredemeyer et al. 2021) where the domestic cat haplotype exhibited features of chromatin organization characteristic of the inactive X, whereas the alternative Asian leopard cat haplotype maintained a structure analogous to the active X of male fibroblast cells. We hypothesized that this reflects skewing of X inactivation in the domestic cat haplotype of the female Bengal F1 hybrid, a phenomenon previously described in interspecific rodent crosses that were used to generate phased Xa and Xi Hi-C maps (Deng et al. 2015; Darrow et al. 2016). Thus, we used the domestic cat and Asian leopard cat X chromosomes to represent the female Xi and Xa state, respectively (supplementary fig. 18, Supplementary Material online). We detected depletion of A/B compartmentalization between the Xa and Xi state, akin to mouse and human (Rao et al. 2014; Darrow et al. 2016), in addition to degeneration of both topologically associated domains (TADs) and formation of a bipartite structure (supplementary fig. 19, Supplementary Material online). Although Hi-C data from domestic cat pachytene spermatocytes and round spermatids did not reveal formation of an analogous bipartite structure, we did observe depletion of intrachromosomal interactions and TADs as spermatogenesis progressed, a feature originally described in the mouse (fig. 7*A*) (Alavattam et al. 2019; Patel et al. 2019). X chromosome A/B compartmentalization showed clear changes across stages of domestic cat spermatogenesis, suggesting that broader features of large-scale nuclear organization may be conserved between silenced X chromosomes in both sexes (fig. 7*B*).

### DXZ4 Is a Rapidly Evolving Macrosatellite

The hypervariable nature of *DXZ4* observed here between different cat species, and described in humans, implicates copy number variability as a likely source of genetic incompatibility between closely related species. We postulate that this interspecific variation in copy number drives the aforementioned shift in methylation and transcriptional profiles across *DXZ4* in the testes of sterile hybrid males. Coincidentally, copy number dependent misregulation has been reported previously in another macrosatellite *D4Z4*. Like *DXZ4*, *D4Z4* maintains an inactive, hypermethylated state when surrounding chromatin is otherwise hypomethylated and active (Chadwick 2009). However, this state is reversed when the total number of satellite repeat units falls below a certain threshold. A shift from hyper- to hypomethylation as a result of fewer repeat units leads to upregulation of genes within each *D4Z4* repeat unit, resulting in facioscapulohumeral muscular dystrophy (Hewitt et al. 1994; Van Overveld et al. 2003).

Copy number dependent regulation of the *D4Z4* macrosatellite is just one example of a conserved regulatory mechanism referred to as repeat-induced gene silencing (RIGS) (Ogaki et al. 2020). The process of RIGS, originally observed in *Drosophila and Arabidopsis* transgenes, was proposed to protect the genome from transposons and other foreign sequences that rapidly duplicate and disperse throughout the genome (Assaad et al. 1993; Henikoff 1998). Studies in mouse revealed that decreasing the copy number of certain transgenes would substantially increase the level of expression per copy, whereas increasing copy number would lead to increased suppression of the transgene (Garrick et al. 1998). Misregulation resulting from copy number variation might provide an intriguing general mechanism for how interspecific divergence might destabilize other complex macrosatellite regions with roles in developmental or reproductive processes.

Until the release of the human X chromosome telomere-to-telomere assembly (Miga et al. 2020), the mouse was the only mammal where *DXZ4* was fully represented in the genome assembly. This is likely due to the smaller and less complex structure of murine *Dxz4*, which is composed of 7 repeat monomers of varying length (3.8 and 5.7 kb), whereas human *DXZ4* contains between 12 and 120 repeat monomers of very similar length (3.0 kb) (Tremblay et al. 2011; Horakova, Calabrese, et al. 2012; Schaap et al. 2013) (supplementary fig. 20, Supplementary Material online). Despite this dramatic structural divergence, *DXZ4* in both human and mouse maintains its putative organizational role forming the bipartite structure of the Xi generated by XCI (McLaughlin and Chadwick 2011; Horakova, Moseley, et al. 2012). Our assemblies of *DXZ4* loci from two cat species surprisingly revealed that while the felid structure was much more similar to human, it also differed significantly from both human and mouse orthologs by being composed of two divergent tandem repeats separated by a spacer sequence. The felid RA unit is likely orthologous to the human *DXZ4* repeat unit based on greater sequence identity and similar CTCF-binding motif patterns. The second repeat array in felids, RB, is absent in both the human and mouse assemblies. The number and orientation of inter-repeat CTCF-binding motifs in the mouse ortholog did not resemble those in either felid repeat array.

The compound nature of *DXZ4* repeat arrays in cats complicates our previous hypothesis that copy number incompatibility is the likely underlying cause of misregulation in the testes of sterile Chausies. In silico copy number estimates indicate that RA expansion in Jungle cat matches the expectations of RIGS, but RB does not. This relationship is especially pronounced in established breed domestic cats like the Egyptian Mau, which were used in founding the Chausie hybrids used in the study. In Jungle cat, copy number of RA is always larger than RB, with the situation being reversed in domestic cat. Additionally, RA alone showed significant methylation and transcriptional differences between sterile and fertile Chausies, further supporting our hypothesis that variation at RA contributes to the hybrid sterility phenotype in Chausies.

We postulate that misregulation of RA could result in failure of MSCI through a number of mechanisms. The first implicates incompatibility between transcripts produced by the Jungle cat *DXZ4* allele and regulatory machinery utilized within the domestic cat background. Our investigation of Jungle cat and domestic cat transcript annotations revealed minimal divergence between conserved exons of Jungle and domestic Repeat A and B Spanning Transcripts (*RAST*, *RBST*) orthologs, however, we also identified unique Jungle cat isoforms that differ in exon usage (*RAST*) and length (*RBST*). Because *RAST* was downregulated in sterile backcross Chausies, it is possible that the Jungle cat *DXZ4* allele is either failing to express *RAST* at all, or expressing the exon variable *RAST2* isoform that is potentially ineffective within hybrids with a predominantly domestic cat genomic background. This hypothesis provides a plausible explanation for *DXZ4* hypermethylation within the sterile Chausie, as silencing of the orthologous human *DANT1-ATT* isoform was also shown to correspond with a hypermethylated, constitutive heterochromatin state in human embryonic stem cells (Figueroa et al. 2015).

A second conceivable mechanism for failure of MSCI through RA misregulation is through epigenetic interference between DNA bound methyl groups and CTCF proteins (Filippova 2007). Hypermethylation of RA in hybrids could prevent binding of CTCF proteins to sequence motifs present within each repeat unit, a situation comparable to *DXZ4-*embedded CTCF motifs on the human Xa (Chadwick 2008). Although inspection of methylation data revealed a significant peak in MF across the CTCF-binding motif of RA, future comparisons between sorted germ cells from fertile and sterile Chausies that apply ChIP-seq and Hi-C experiments are required to properly test these different hypotheses.

## Conclusions

We identify the *DXZ4* macrosatellite as a novel mammalian X-linked candidate hybrid-sterility locus in an interspecific cross between domestic cats and Jungle cat, two species that diverged ∼3 − 4 Ma (Johnson et al. 2006; Li, Davis, et al. 2016; Li, Hillier, et al. 2016; Li et al. 2019). *DXZ4* is a compelling candidate hybrid sterility locus based on its structural complexity, known role in sex chromosome silencing, and peculiar biology. The rapid mutation rate of *DXZ4* fits the theoretical requirements of a “speciation gene,” where evolutionarily labile satellite DNA represents a common template upon which genetic incompatibilities rapidly arise. The implication that rapid evolution of sex-linked satellite elements may contribute to infertility and inviability, and thereby promote speciation, has received support in previous studies of *Drosophila* interspecific hybrids (Bayes and Malik 2009; Ferree and Barbash 2009; Ferree and Prasad 2012). The satellite-encoded hybrid sterility gene *OdsH* from *Drosophila mauritiana* ChrX acts as a sterilizing factor in male *Drosophila simulans/mauritiana* hybrids by associating with the heterochromatin of the *D. simulans* Y chromosome, whereas the *D. simulans* ortholog does not (Bayes and Malik 2009). Similarly, it was predicted that interspecific satellite divergence in mammals would also play a significant role in reproductive dysfunction and speciation (Yunis and Yasmineh 1971).

The discovery of the unique compound *DXZ4* repeat structure in felids immediately raises many questions regarding its function during XCI, and potentially MSCI. Whereas RB is absent in humans and mice, what is its significance in felids? Is RB shared by all cat species? Is the possession of both RA and RB the ancestral or derived state for placental mammals? In this study, we limited our investigation of *DXZ4* transcription to expression in male germ cells. To fully assess the function of both *DXZ4* repeat arrays, future work will require transcriptional analysis of the locus in other cell types, particularly female embryonic stem cells undergoing XCI. It is also unclear how differing CTCF-binding motifs of each repeat unit in felids might correspond to the long-range physical interactions formed between X-linked MSR loci, *DXZ4*, *FIRRE*, and *ICCE* in human XCI. Although conservation of the bipartite structure on the felid Xi suggests that these *DXZ4* interactions are maintained, our Hi-C resolution was incapable of detecting superloops between the MSR loci in cats and will require improved Hi-C resolution or more sensitive, targeted 4C-approaches. Additional comparative sequencing and functional studies are necessary to more fully understand the full repertoire of roles played by *DXZ4* in mammalian biology.

## Materials and Methods

### Chausie Hybrids

The Chausie cat breed was originally established by hybridization between the domestic (*Felis catus*) and Jungle cat (*Felis chaus*), two species that diverged ∼3 Ma (Li et al. 2019). The Chausie is derived from the least diverged interspecific cross of the previously described breeds (Li, Davis, et al. 2016; Li, Hillier, et al. 2016; Li et al. 2019). Like Bengals and Savannahs, early generation hybrid Chausies follow Haldane’s rule by exhibiting hybrid male sterility and require F1 females to be backcrossed to fertile male domestic cats, or to late generation fertile backcross Chausie males, as explained below. The number of generations required for rescue of male fertility is dependent on parent species divergence times and is correlated to the percentage of wildcat ancestry maintained in male individuals (Davis et al. 2015; Allen et al. 2020). After two to three generations, litters begin producing fertile males, allowing breeding between hybrid individuals. Today’s Chausie breed represents a population of breeding hybrid males and females from generations spanning F1s (typically females) to hybrids more than six generations past F1. Later backcross generation hybrid males, typically F3−F5, exhibit variable fertility between individuals. Determination of fertility for all Chausies was performed using one or both of two methods: Breeding records and histopathology as described in Davis et al. (2015). Sterility was defined as repeated, confirmed matings with multiple proven breeder females over 1 or more years with no conception, whereas fertile individuals were defined by documented productive breeding with validation via pedigree records.

### Histopathological Evaluation of Backcrossed Chausie Testes

Histopathological evaluation was performed on testes and epididymides from sexually mature males that underwent orchidectomy. Testes were laterally bisected and stored in Bouin’s fixative and later transferred to formalin. Testes and epididymides were embedded in paraffin, sectioned, and stained with H&E. Histology was evaluated to determine the presence/absence of germ cells, stage of meiotic progression, and the presence/absence of normal sperm. Fertile individuals possessed seminiferous tubules and caput epididymides with large numbers of sperm displaying normal morphology. Histological data were available for all individuals utilized in RNA-Seq analyses.

### RNA-Seq and Differential Expression Analysis

Testes from two sterile and two fertile backcrossed Chausies were obtained from sexually mature cats that underwent orchidectomy. Testes were bisected and seminiferous tubules isolated. RNA and DNA were extracted using Trizol Reagent (Applied Biosystems 2010). Extracts were assessed for quality using the Agilent 2200 Tape Station System. Extracted RNA was used to generate both RNA-depleted and small RNA libraries (NEXTFLEX, Perkin Elmer) and sequenced on the Illumina HiSeq 2500 to obtain an average of 40 million paired end reads per sample. Examination of sequencing data included *FastQC v0.11.8* (Andrews 2010) followed by adapter trimming using *Trim Galore! v0.6.4* (Babraham Bioinformatics 2020). Trimmed reads were mapped to the single haplotype domestic cat assembly Fcat_Pben_1.0_maternal_alt (Fca-508: GCA_016509815.1) using *STAR v2.7.7a* (Dobin et al. 2016) with default settings. We used *SAMtools v1.9* (Li et al. 2009) to process the alignment into a sorted bam file for downstream analysis. Raw read counts were calculated from bam files using *HTSeq-count v0.13.5* (Anders et al. 2013). For comparison of genes expressed between sterile and fertile hybrids differential expression analysis was performed using the Bioconductor package *edgeR v3.32.1* (Robinson et al. 2010; McCarthy et al. 2012). For assessing X chromosome misregulation in sterile testes, we first established an expression profile for fertile testes and identified upregulated genes based on whether or not they were previously expressed in fertile testes. Chromosome-wide misexpression was tested for significance using a chi-squared test. Difference in upregulation between the X and autosomes was tested for significance using Fisher’s exact test. Statistical tests for chromosomal enrichment of DE genes were performed based on expectations generated using Markov chain Monte Carlo simulations. Gene Ontology enrichment analysis for biological processes was performed using *PANTHER v.14* (Mi et al. 2019; Ashburner et al. 2000).

### GWAS and Fine Mapping

A binary case−control GWAS was performed on a cohort of 23 fertile and 16 sterile backcross Chausie hybrids that were genotyped on the Illumina 63K Feline SNP array. Hybrids possessing a genotype call rate <0.9 were removed from further study. We searched for marker-based-associations meeting or exceeding the Wellcome Trust recommendations (*P*_uncorrected_ = 5 × 10^−5^; -log_10_*P *=* *4.30) (Wellcome Trust Case Control Consortium 2007). Notably, this significance threshold is conservative considering the polygenic nature of hybrid sterility and the modest SNP density of the Illumina feline SNP array, with the Wellcome Trust recommendations developed for a much higher density SNP array (Human Affymetrix 500 K GeneChip; see Wellcome Trust Case Control Consortium 2007). All marker-based association analyses were carried out using a mixed linear model, as described and implemented in EMMAX (Kang et al. 2010; Segura et al. 2012), and were executed in the SVS environment (Golden Helix, Version 7.7.6) as described (Davis et al. 2015; Seabury et al. 2017).

Fine mapping of a critical interval was performed using bi-allelic SINE INDEL marker assays that distinguish the domestic cat from Jungle cat X chromosome. SINE INDELs homozygous in the reference female domestic cat and homozygous null in the female Jungle cat pseudoreference were identified by aligning Jungle cat Illumina short reads (SRA accession number: SRX1058146) to the v8.0 “Cinnamon” reference genome (Li, Hillier, et al. 2016). Thirty-five candidate fixed SINE insertions in the domestic cat were used to design primers for PCR-testing (following Murphy and O’Brien 2007) in ten random-bred domestic cats and eight Jungle cats (six captive zoo animals from Central Asia and two from Thailand) and resolved on 2.5% high-resolution agarose gels. A final set of 27 SINE markers that were informative for ancestry inference [i.e., homozygous insertions in all domestic cats and absent (null) in all Jungle cats] were used to assay hybrid backcross Chausies.

### Jungle Cat Genome Assembly

To create a Jungle cat genome reference assembly, we extracted DNA from an early passage, primary fibroblast cell line, derived from a captive-born male Jungle cat, housed originally at the Blijdorp Zoo, Rotterdam. To resolve assembly issues resulting from large, repetitive, and highly polymorphic regions, a male individual was selected to generate an effectively haploid (excluding the pseudoautosomal region) X chromosome assembly. The same cell line was used to generate PacBio, Illumina, and Hi-C libraries. The PacBio SMRT library was sequenced on the PacBio Sequel platform, resulting in 7,594,421 reads with an N50 subread length of 25.89 kb, corresponding to ∼50× coverage relative to the length of the domestic cat reference assembly, felCat9 (Buckley et al. 2020). Illumina sequence libraries were generated using the NEB-Ultra II kit and sequenced on the Illumina NovaSeq6000, yielding ∼33× coverage, and were used for polishing of the PacBio contigs. Sequence adapters were trimmed using *Trim Galore!* and read quality assessed using *FastQC*. Hi-C libraries were prepared following the Ramani (2016) protocol for in situ DNase Hi-C. Libraries were sequenced on the Illumina NovaSeq6000, yielding ∼1.5 billion paired end reads (180× Coverage). Hi-C reads were trimmed using *Trim Galore!* with additional commands *–clip_R1 10 –clip_R2 10 –three_prime_clip_R1 15 –three_prime_clip_R2 15* selected to remove 10 and 15 bp from the 5′ and 3′ end of each read, respectively. Contig assembly was performed with *NextDenovo v2.2-beta.0* (github: Nextomics/Nextdenovo) with the configuration file (.cfg) altered for inputs: *minimap2_options_raw = -x ava-pb*, *minimap2_options_cns = -x ava-ont*, and *seed_cutoff=* 13944.

### Mitochondrial Genome Assembly

Prior to polishing raw contigs with short reads, we screened the assembly using BLAST to detect the presence of a complete Jungle cat cytoplasmic mitochondrial (*cymt*) sequence. We performed this check because previous studies had verified the presence of nuclear mitochondrial (*numt*) sequences within the domestic cat and *Panthera* nuclear genomes (Lopez et al. 1996; Kim et al. 2006). Despite residing in distinct cellular environments, *numt and cymt* sequences are highly similar, with *cymt* reads being far more abundant due to higher copy number per cell. Failure to account for *numt* sequence during the assembly polishing step with short-read data would potentially result in conversion of *numt* to *cymt* sequence if a more similar *cymt* “bait” sequence is excluded from the assembly (Rhie et al. 2020). Our BLAST analysis of the Jungle cat assembly identified a single significant hit to contig ctg000098, a chimer of tandemly duplicated jungle cat *cymt* sequences and chromosome D2, which harbors the domestic cat *numt* sequence. (Antunes et al. 2007). To isolate the full-length assembly of the Jungle cat *cymt* sequence we performed an alignment between ctg000098 and the previously published Jungle cat *cymt* sequence (Li, Davis, et al., 2016; Li, Hillier, et al. 2016) using LastZ (Harris 2007). We then used changes in percent identity across the alignment to distinguish and extract the jungle cat *cymt* sequence from surrounding chromosome D2 *numt* sequence.

### Contig Polishing, Purge-Dups, and Quality Control

We polished the raw assembly contigs with *NextPolish v1.3.0* (Hu et al. 2020), using the *NextDenovo* corrected long reads, and Illumina short reads. Notable changes to the *NextPolish* configuration file included: *genome_size=auto*, and *task=best*, which instructs the program to perform two iterations of polishing. We used default settings for both *sgs and lgs* read mapping options except for indicating PacBio input with *minimap2_options= -x map-pb*. Following polishing, *purge-dups v.1.0.1* (Guan et al. 2020.) was used to remove haplotigs and smaller, low coverage contigs. Basic assembly stats were generated using *QUAST v5.0.2* (Mikheenko et al. 2018) with the minimum contig length set to *–m 1* and the *–fast* run option selected. To assess genome completeness, *BUSCO v4.0.6* (Simão et al. 2015) was run using the *–m* genome setting with *–l mammalia_odb10* database selected (9,226 single copy genes). Visual assessment of the haploid assemblies was performed through alignment to the single haplotype domestic cat assembly Fca-508 using *nucmer* (*mummer3.23* package; Marçais et al. 2018) with default settings. The resulting delta file was used to generate a dot plot visualized in *Dot: interactive dot plot viewer for genome-genome alignments* (DNAnexus).

### Y Chromosome Contig Identification and Isolation

To identify Y chromosome contigs within the Jungle cat assembly, we used two parallel approaches. The first is based on mapping female Jungle cat Illumina reads to the male Jungle cat PacBio contigs. This approach relies on the expectation that female reads will lack Y chromosome sequence and thus allows for the selection of contigs based on zero or limited read coverage across their length. Illumina sequence reads from a female Jungle cat were previously generated by Li, Davis, et al. (2016) and Li, Hillier, et al. (2016), accession number SRR2062187, and aligned to the contigs using *bwa mem v0.7.17* (Li et al. 2009) with default settings. The alignment output was piped into *Samtools v1.9* (Li et al. 2009), where it was converted into bam format, sorted, and indexed. To annotate coverage across the assembly we used the *genomecov* tool of the *BEDTools suite v2.29.2* (Quinlan and Hall 2010) with the −*d* (for per base coverage) and −*bga* (for regional coverage as bedgraph) options selected. The results were output in bedgraph format and all contig nucleotide positions identified as having a female read coverage threshold of 15×, 10×, 5×, or 0×, and extracted into separate lists. For each coverage list *R v.3.5.1* (R Core Team) was used to calculate the percent of positions in each contig at, or below, the coverage threshold. This was done by summing the total number of positions annotated from *BEDTools* for each contig, and dividing by the contigs total length.

Next, to begin screening for Y contigs, we extracted contigs with 70 − 100% of their nucleotide positions within the threshold coverage. The first step in determining this cutoff was to identify Y contigs using known domestic cat Y chromosome sequences. To do this, we used NCBI’s basic local alignment search tool *(BLAST) v2.9.0* (Altschul et al. 1990) command line application with options *-culling_limit 1, -evalue 1e-25, and -perc_identity 85* specified to align domestic cat mRNA sequences (Murphy et al. 2006; Pearks Wilkerson et al. 2008; Li et al. 2013) and ampliconic Y chromosome BAC clones (Brashear et al. 2018) to the Jungle cat assembly custom BLAST database. Because the Jungle and domestic cat diverged fairly recently (∼3 Ma) (Li, Davis, et al. 2016) the *megablast* algorithm was used to generate alignments. *GNU parallel* (Tange 2011) was used to BLAST multiple queries simultaneously.

Finally, the average percent positional contig coverage identified by single copy mRNA sequences was averaged and ±25% added to determine the percent position coverage cutoff. This threshold was further justified by plotting a histogram for the data where we observed a bimodal distribution for the number of contigs either lacking or possessing female read coverage across most of their lengths. This result suggests the cutoff was sufficient for capturing Y sequence contigs whereas avoiding autosome/X-linked contigs. Finally, *megablast* was used to align contigs with 70% of their nucleotide identity possessing 15× coverage or less to the felCat9.0 assembly (Buckley et al. 2020). For this step, only the top BLAST hit was output using command line options *-num_alignments 1*, *-max_hsps 1*. To avoid false hits to repetitive elements both the felCat9.0 custom database and Jungle cat contigs were repeat-masked using *RepeatMasker v4.0.9* (Smit et al. 2013 − 2015) with default settings and option *-species felidae* selected. Using these results, we manually selected additional contigs presumed to be Y linked based on a combination of alignment percent identity to fca-9.0 autosomal sequences, percent nucleotide positions covered by female reads, and total sequence length. All contigs identified in this way were merged into a single list and removed from the assembly using *seqTK subseq v1.3* (Heng 2018). These extracted sequences were then scaffolded using Hi-C libraries using two different approaches. The first was a more automated approach using *SALSA v2.2* (Ghurye et al. 2017, 2019) with reads preprocessed and mapped following the esrice slurm-hic pipeline run manually (github: esrice/slurm-hic). *SALSA* was run on the resulting bedfile with default options except *-e DNASE, -m yes*. This was followed by manual curation of Hi-C read mapping using *Juicer v1.5.7* (Durand, Shamim, et al. 2016), followed by scaffolding with *3d-dna v180922* (Dudchenko et al. 2017) and *Juicebox Assembly Tools* (*JBAT*, *Juicebox v1.11.08*) (Durand, Robinson, et al. 2016).

### Scaffolding

Polished contigs (excluding those removed by purge-dups or identified as Y) were first scaffolded using Hi-C data and *SALSA v2.2* with parameters *-e none -m yes.* Inspection of the SALSA scaffolds was performed using *QUAST*, *nucmer*, and *Juicebox*. Following *SALSA*, *RagTag v1.0.1* (Alonge et al. 2019) was used to localize scaffolds to their respective position in the chromosome length single haplotype domestic cat assembly Fca-508. Selected *RagTag* parameters included *–remove-small*, *–f 10000*, and *–j unplaced.txt*, a text file of scaffolds for *RagTag* to ignore based on their small size and identification as repetitive sequence in the *nucmer* alignments. *RagTag* scaffolds were manually inspected with Hi-C maps generated using *Juicer v1.5.7* with option *-s none* selected for compatibility with DNase Hi-C libraries. Maps were visualized using *Juicebox v1.11.08 and Juicebox Assembly Tools* with scripts from *3d-dna v.180922*.

### Genome Annotation

Repetitive sequence annotation was performed with *RepeatMasker v4.0.9* with -*excln and -species cat* selected to identify and annotate repetitive regions of both genomes while ignoring gap sequence. To estimate indel rates and quantify repeat expansion and contractions we ran *Assemblytics v1.2.1* (web-based) (Nattestad and Schatz 2016) with a unique sequence length requirement of 10,000 on nucmer alignments between domestic single haplotype assemblies. Because of the high sequence similarity between the domestic and Jungle cat genomes, we used *Liftoff v1.4.2* (Shumate and Salzberg 2020) to perform an annotation lift over between the current felCat9 reference assembly (Buckley et al. 2020) with single copy Y chromosome sequence (Li et al. 2013) and the Jungle cat de novo assembly. Default parameters were used for all arguments except for calling *-copies* with *-sc 0.95* to identify extra copies of genes not previously annotated in felCat9.

### DXZ4 Repeat Unit Analysis and In Silico Copy Number Estimation

Identification and isolation of *DXZ4* repeat units was performed manually using GC content traces, CTCF-motif annotations, and self-self dotplots for the region using *Geneious Prime v2021.0.3*. CpG islands were annotated using *Geneious CpG Plugin* based on the Hidden Markov Model Forwards/Backwards algorithm described in Durbin et al. (1998). CTCF motifs were annotated using the *Geneious Annotate & Predict* tool with a sequence motif of GAGTTTCGCTTGATGGCAGTGTTGCACCACGAAT, based on the Horakova, Calabrese, et al. (2012) conserved CTCF motif logo, with the most prevalent nucleotide representative of each position. A max mismatch of 13 was selected to allow for interspecific ambiguity within the motif. CTCF sites annotated using this method corresponded to the approximate location within human *DXZ4* repeat units originally described by Chadwick (2008). CTCF binding motif annotations were further confirmed using the *MEME Suite v5.3.3 FIMO* (Bailey et al. 2009; Grant et al. 2011) tool with the following motif input (using the IUPAC nucleotide ambiguity code): VVNYTYYKSTKGRYGGCRVYRBWGYHCYRSVAAT. Once annotated and extracted, independent repeats were aligned using the *Mafft Multiple Aligner v1.4.0*. Neighbor joining consensus trees were generated using the *Geneious Tree Builder* plugin and maximum likelihood trees generated using the Geneious *RAxML v8.2.11* (Stamatakis 2014) plug-in with nucleotide model: *GTR GAMMA*, Algorithm: *Rapid hill-climbing* and Replicates: *500* selected. Mean within and between group distances for masked (10% gaps masked) *DXZ4* repeat unit alignments were calculated using *Mega-X v10.0.5* (Kumar et al. 2018).

In silico estimations of copy number were performed using short read mapping across collapsed tandem repeats (Lucotte et al. 2018). A representative unit from each of the *DXZ4* Repeat A and Repeat B arrays was selected from each of the three assemblies (domestic, Jungle, and Asian leopard cat) based on pairwise identity visualized using a neighbor joining tree and inserted in place of the full repeat array in the X chromosome of each assembly. We also included the first (most proximal) copy of RA (RA-1) due to its divergence from other RA units. Illumina short-read data from 12 domestic cats (representing both outbred and established breeds), 1 Jungle and 1 Asian leopard cat (supplementary table 8, Supplementary Material online) were mapped to their respective *DXZ4* modified genome assemblies using *bwa mem v0.7.17* (Li et al. 2009). Male individuals were selected when available to avoid confounding of copy number estimates across two haplotypes, which occurs in females. Alignment files were processed using *samtools fixmate*, *sort*, *markdup*, and *view* with *-q 20* and *-bh* specified (*v.1.10*; Li et al. 2009). Coordinates for the collapsed *DXZ4* regions and the single copy control gene *DMD* used in Lucotte et al. (2018) were recorded in a BED file and used to calculate the mean across feature coverage using *bedtools coverage v2.30.0* with *-mean* called (Quinlan and Hall 2010)*. DMD* was verified as single copy in the three genome assemblies using the lift over GFF file. Average coverage across the entire genome of each individual was generated from the filtered and sorted BAM file using *bedtools genomecov* with *-d* selected, and used to calculate copy number across each repeat unit and *DMD*. We observed an average coverage of 0.5 across *DMD* for all X hemizygous male individuals, as expected. Female individuals also exhibited the expected *DMD* coverage of 1.0 and had repeat estimates divided by 2 to account for diploidy.

### Reduced Representation Bisulfite Sequencing

We obtained testes from six felids (fertile domestic cat males = 2, Chausie backcross males = 4, two fertile, two infertile) and two domestic cat germ cell populations (pachytene spermatocytes = 1, round spermatids = 1), which were sequenced at an average depth of 22.2- and 20.2-fold for autosomes and the X chromosome, respectively. Chausies were selected on the basis of having near-identical estimated % Jungle cat ancestries (supplementary table 9, Supplementary Material online). Genomic DNA libraries were prepared with an RRBS approach using the Msp1 restriction enzyme (Boyle et al. 2012) and the NEBNext sample preparation kit (New England Biolabs) (supplementary table 10, Supplementary Material online). Each library was spiked with 1 ng of enterobacteria phage lambda DNA as a nonmethylated internal control for estimating bisulfite (BS) conversion (e.g., Lea et al. 2015). For all purification or size selection (100 − 400 bp) steps, we used AMPure XP beads (Beckman Coulter). Fragmented DNA was treated with bisulfite to convert unmethylated cytosines following the low DNA input protocol in the Qiagen EpiTect Fast Bisulfite Conversion kit (Qiagen USA). We enriched the converted DNA for adapter-ligated fragments with 12 cycles of PCR amplification and MyTaq Mix (Bioline Inc). Simultaneously, unique sequence tags were included in the amplification to barcode each library to enable pool of seven samples per lane of single-end (1 × 100nt) sequencing on an Illumina NovaSeq 6000.

Sequence pools were demultiplexed based on perfect sequence matches between expected and observed barcode sequence tags. We trimmed reads to remove low quality bases (*Q* < 20), clipped remnant adapter sequences, and discarded reads that were <20 bp in length using cutadapt 1.8.1 (Martin 2011). To prevent loss of signal due to multimapping across the *DXZ4* locus we collapsed the Fca-508 genome assembly RA and RB repeat arrays into a single representative repeat unit for each array. The modified Fca-508 assembly was subsequently prepared with bowtie2 in BS-Seeker2 for read lengths bounded from 50 to 500 bp (Chen et al. 2010; Langmead 2010; Langmead and Salzberg 2012). We aligned processed reads to the built reference with bowtie2 and called methylation in BS-Seeker2. We calculated the MF per cytosine as the proportion of methylated cytosines from the total read depth per site (Chen et al. 2010). Bisulfite conversion efficiency was estimated by mapping each genomic library to the 48,502-bp phage lambda linear genome (NC_001416.1) and assessing the MF of the lambda-mapped data from cytosines with at least 10× sequence coverage. Conversion rates were estimated as [1 − average MF across the phage lambda genome]. We used the unite function in R v3.6.0 (2019) MethylKit (Akalin et al. 2012) package to apply a coverage filter to retain cytosines with a depth of coverage between 10× and below the 99.9% percentile. We included all methylation motifs (CG, CHH, and CHG) for analysis. We constructed a methylation matrix across all eight samples. To assess library quality, we used the *prcomp* function in *R v3.6.0* (R Core Team 2019) to conduct a PCA.

### X Chromosome Candidate Region Analysis

We further scrutinized the *DXZ4* gene region (94,183,053 − 94,228,160 Mb) to evaluate methylation trends by plotting simple moving averages with the geom_ma function from tidyquant package. We also constructed sliding window plots using a cubic smoothing spline with the R packages GenWin and pspline to fit per-cytosine MF estimates (Craven and Wahba 1978; Beissinger et al. 2015). We used the generalized cross-validation smoothing (λ) method to identify the inflection points of the spline to define the window boundaries. We averaged MF in the RA and RB *DXZ4* regions across groups of felids and conducted simple *t* tests of differences per cytosine in sliding windows with 20 cytosines per window and a 10-cytosine step. We filtered the data to include only cytosines sequenced in all eight felid samples to reduce intrawindow disparities in sample size.

### Domestic Cat Sorted Germ Cell RNA-Seq

Testes for germ cell sorting were collected from adult male domestic cats that underwent orchidectomy at the Texas A&M small animal hospital. Assumptions of fertility were based on maturity of animal and relative testes size. Testes were collected and immediately placed in cell culture media w/FBS prior to cell sorting. Target populations of pachytene spermatocytes and round spermatids were collected using the STA-PUT method of sedimentation velocity (Go et al. 1971; Wang et al. 2001), snap frozen in liquid nitrogen and stored at −80 °C. Purities of recovered populations of pachytene spermatocytes and round spermatids were ≥90% based on morphological analysis under phase optics.

For each germ cell population both RNA and DNA were extracted using Tri-Reagent (Applied Biosystems). An aliquot of DNA was used for RRBS sequencing and extracted RNA was used to create two different RNA-Seq libraries from each of the sorted populations. Additionally, RNA-Seq libraries were generated from whole seminiferous tubules of two domestic cats and one Jungle cat. For each sorted germ cell population, a technical replicate RNA-Seq library was generated for both protocols. The first RNA-Seq library was preprocessed using the NEBNext rRNA Depletion Kit and subsequently converted into an RNA-Seq library using the NEBNext Ultra Directional RNA Library Prep Kit (Illumina). The second library was generated using the NEBNext Multiplex Small RNA Library Prep Set (Illumina). The rRNA-depleted libraries were sequenced on the Illumina HiSeq 4000. The smallRNA library was first size selected for 105 − 160 bp fragments using the Pippin-Prep and sequenced on the Illumina HiSeq 2500v4 in rapid mode for generation of 50-bp single-end reads. Sequencing data was checked for quality and postprocessed using *FastQC and Trim Galore!*.

### RNA-Seq Read Mapping and Analysis

Trimmed reads were mapped to the single haplotype domestic cat (Fca-508) and de novo Jungle cat assembly (FelChav1.0) using *STAR* with default settings and *–outSAMstrandField intronMotif* to enable downstream compatibility with *Cufflinks*. Technical replicates were merged using *samtools merge* prior to annotation. *Cufflinks v2.2.1* (Trapnell et al. 2012) was used to generate de novo annotations previously missing from the felCat9 lift over annotation as a result of germ cell specific expression or increased sensitivity to detection of lowly transcribed small and lncRNAs afforded by our RNA-Seq library protocols. SmallRNA libraries were annotated using *ShortStack v 3.8.5* with *-dicermax 31 and -mincov 0.5 rpm* specified (Axtell 2013). Transcripts, annotations, and read alignments were visualized and assessed using IGV and Geneious.

### In Situ DNase Hi-C

Hi-C libraries were prepared following the Ramani (2016) protocol for in situ DNase Hi-C. Fibroblasts from male domestic cat and sorted germ cell populations were fixed, converted into Hi-C libraries and sequenced to ∼50× coverage. Previously published Hi-C data haplotype-phased from an F1 Bengal (Bredemeyer et al. 2021) suggested skewing of XCI towards the domestic cat X, based on comparisons between the haplotyped X chromosomes. The domestic cat X Hi-C map exhibits features’ characteristic of an inactive X, whereas structural conformation of the Asian leopard cat X was more similar to autosomes and the active X of male fibroblast cells. Thus, we used the domestic cat X and Asian leopard cat X to represent the female Xi and Xa state, respectively. All cell types were selected because they were representative of the X chromosome in a haploid state. The domestic cat Xi, pachytene, and round spermatid cell types were selected to observe the inactive or partially inactive X chromosome in the two sexes. For comparison, male fibroblasts were selected to represent a single haplotype active X state. Maps were generated using *Juicer v1.5.7* with *-s none* selected for compatibility with reads from libraries generated using DNase as the fragmenting enzyme. Hi-C maps were visualized using *Juicebox v1.11.08*. Finer resolution Pearson’s plots were generated using *juicer-tools pearsons* with *-p KR* selected for normalization and *BP 250000* selected to set a bin size of 250 kb.

## Supplementary Material

Supplementary data are available at *Molecular Biology and Evolution* online.

## Supplementary Material

msab274_Supplementary_Data
