# Supplementary material for: Rapid Macrosatellite Evolution Promotes X-Linked Hybrid Male Sterility in a Feline Interspecies Cross
Source: Mol Biol Evol. 2021 Sep 14;38(12):5588–609. doi: 10.1093/molbev/msab274 (PMC8662614; doi:10.1093/molbev/msab274)
Supplement: msab274_Supplementary_Data [file msab274_supplementary_data.pdf]

Supplementary Information for

Rapid macrosatellite evolution promotes X-linked hybrid male sterility in a feline interspecies cross.

Kevin R. Bredemeyer, Christopher M. Seabury, Mark Stickney, John R. McCarrey, Bridgett vonHoldt, and William J. Murphy

Supplemental Discussion. Details of Jungle Cat *de novo* Assembly

Supplementary Figure 1. Per chromosome log fold change of differentially expressed genes
Supplementary Figure 2. *PLS3* expression across multiple tissues
Supplementary Figure 3. Comparison of *DXZ4* region in domestic cat reference genomes
Supplementary Figure 4. Dotplots of sequence gain across *DXZ4* region in each assembly
Supplementary Figure 5. Annotation tracks of complete *DXZ4* regions
Supplementary Figure 6. Felid interspecific alignment of *DXZ4* spacer sequence
Supplementary Figure 7. Sequence design for *DXZ4 in silico* copy number estimation
Supplementary Figure 8. *DXZ4* transcripts in cat and human
Supplementary Figure 9. *DXZ4* transcript alignments between cat and human
Supplementary Figure 10. Interspecific comparison of *DXZ4* transcripts
Supplementary Figure 11. Alignment between domestic and Jungle cat *DXZ4* regions
Supplementary Figure 12. Domestic and Jungle cat transcription across DXZ4
Supplementary Figure 13. Domestic and Jungle cat transcription across DXZ4 RB-1
Supplementary Figure 14. Chausie hybrid transcription across DXZ4
Supplementary Figure 15. PCA from methylation of fertility phenotypes
Supplementary Figure 16. Per chromosome methylation frequency averages
Supplementary Figure 17. Bipartite structure on Xi of cat, mouse and human
Supplementary Figure 18. F1 Bengal phased Hi-C maps
Supplementary Figure 19. Pearson's Xa and Xi maps for cat, human and mouse
Supplementary Figure 20. *DXZ4* structure in human, mouse and cat
Supplementary Figure 21. Jungle cat MT dotplots
Supplementary Figure 22. Jungle cat contig alignment to Fca-508
Supplementary Figure 23. Jungle cat scaffold alignment to Fca-508
Supplementary Table 1. Gene ontology results for X linked upregulated genes
Supplementary Table 2. Raw sequencing output
Supplementary Table 3. *DXZ4* interspecific *P*-distances
Supplementary Table 4. *DXZ4* repeat unit summary
Supplementary Table 5. *In silico* copy number estimates of *DXZ4*
Supplementary Table 6. *DXZ4* differential methylation
Supplementary Table 7. Differential methylation window summary
Supplementary Table 8. SRA accessions of individuals used for *in silico* estimations
Supplementary Table 9. RRBS felid sample meta data
Supplementary Table 10. RRBS mapping summary
Supplementary Table 11. Y linked contigs
Supplementary Table 12. Jungle cat chromosome assembly
Supplementary Table 13. RepeatMasker summary
Supplementary Table 14. Annotation lift over summary
Supplementary Table 15. Jungle cat extra copy genes relative to felCat9.0
Supplementary Table 16. Assemblytics structural variant summary

Supplemental Discussion. Details of Jungle Cat *de novo* assembly

The initial assembly using NextDenovo yielded 174 raw contigs with a single chimeric contig joining chromosomes B3 and E1. 68 haplotigs, duplicate contigs representative of the alternative haplotype, were identified and purged resulting in 106 final contigs. Prior to scaffolding, we identified and isolated 13 Chr. Y contigs totaling 3.48 Mb in length. This was done to prevent incorporation of repetitive Y chromosome contigs into highly similar, but paralogous autosomal regions (Brashear et al., 2018) during scaffolding. The homologous X and Y pseudoautosomal region (PAR) was collapsed into a single contig with ~206 kb of male-specific single copy Y sequence incorporated at the end. Male-specific single copy sequence was removed and manually joined to two additional Y contigs to generate a single Chr. Y scaffold representing the single copy region (SCR) (Supplementary Table 11).

Using BLAST and LASTZ Alignments we were able to identify a complete Jungle cat mitochondrial genome (MT) sequence 17,251 bp in length. The new Jungle cat MT was 598 bp longer than the previous short-read based sequence (16,653 bp; Li et al., 2016) and more similar in length to the reference domestic cat mitochondrial genome (felCat9: 17,009 bp) (Buckley et al., 2020). Self-self dotplot comparisons demonstrated this gain was due to assembly of the highly repetitive control region present in domestic cat but lacking in the published Jungle cat MT sequence (Supplementary Figure 21).

Jungle cat contigs were aligned to the single haplotype domestic cat assembly (Fca-508: GCA_016509815.1) using Nucmer and revealed a majority of chromosome arms were captured in single contigs (70%) with only a single chimeric contig observed prior to scaffolding (Supplementary Figure 22). Scaffolding of the contigs using Hi-C data yielded chromosome length-scaffolds (N50=148.6 Mb). Alignments between the Jungle cat and domestic cat chromosomes revealed large-scale collinearity between the two species, as previously suggested by karyotypic analysis (O'Brien 2020, Atlas of Mammalian Chromosomes, 2nd Edition) (Supplementary Figure 23). A total of 54 gaps remained in the 19 chromosome-length scaffolds (Supplementary Table 12). 32 contigs remained unplaced representing 0.3% of the un-gapped assembly length (11 of these were orthologous to ampliconic regions of the domestic cat ChrY, Brashear et al. 2018). BUSCO analysis revealed that 95% of the 9226 mammalian BUSCOs were represented in the final Jungle cat assembly with most (98%) being complete single-copy. 33.67% of the genome was identified as repetitive (Supplementary Table 13). Gene liftover from the felCat9 reference assembly resulted in the annotation of 19,611 protein coding genes with 203 of these identified as an extra copy relative to the reference (Supplementary Table 14 & 15). Structural variant analysis comparing the Jungle cat assembly to Fca-508 revealed an increase of 5.7 Mb due to repeat expansions as well as insertions of various size (Supplementary Table 16), which accounts for a majority of the 6 Mb un-gapped assembly length difference between the two species.

Supplementary Figure 1. Per chromosome log-fold change (logFC) of genes differentially expressed in the testes of sterile backcross Chausie hybrids relative to the testes of fertile Chausie hybrids. All autosomes show a similar logFC profile while the X chromosome exhibits increased upregulation (logFC= +2.45).

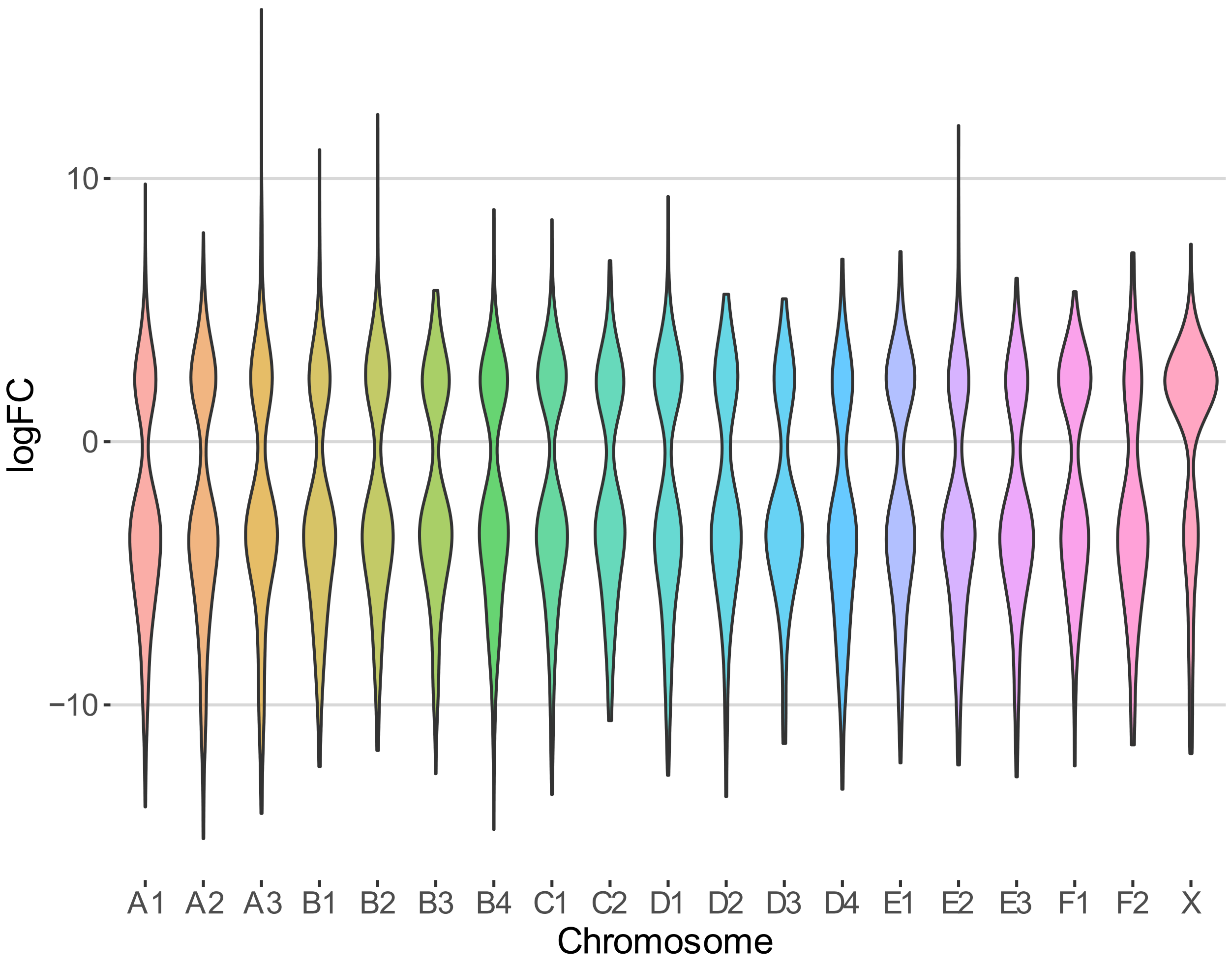

Supplementary Figure 2. RNA-seq data tracks for *PLS3* in the NCBI Genome Browser for domestic cat reference assembly felCat9. The x-axis reflects coordinates across the X Chromosome. Expression tracks for various tissue types are arrayed across the y-axis and are log-scaled. *PLS3* is ubiquitously expressed across tissue types in the domestic cat.

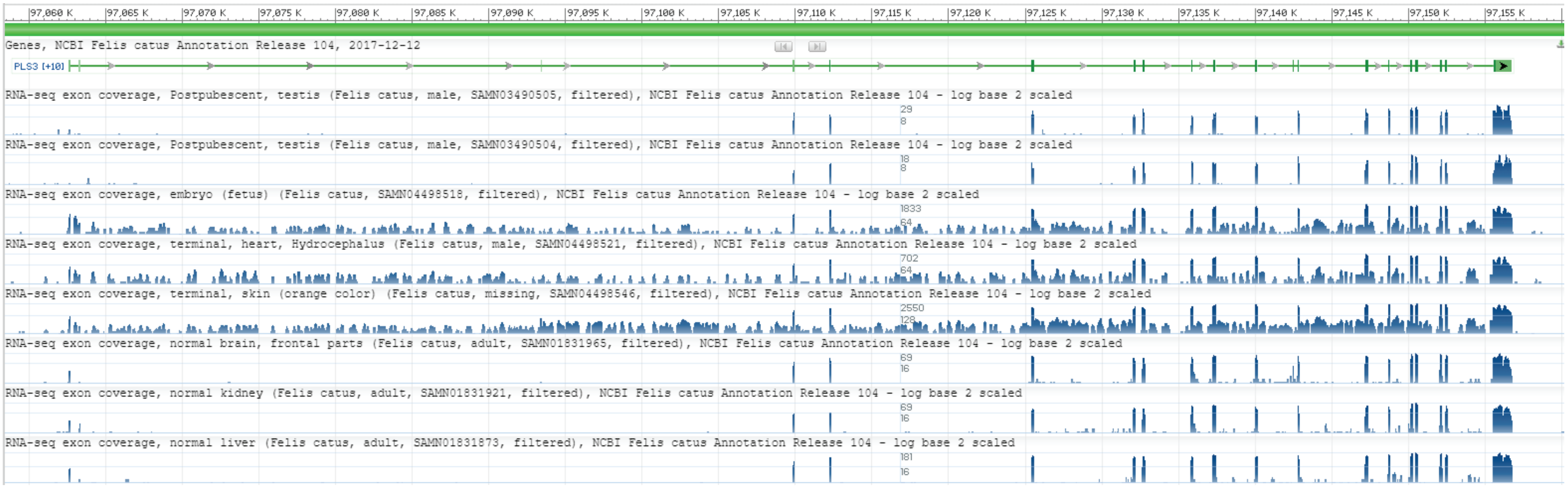

Supplementary Figure 3. Mauve alignment of the *DXZ4* region from three previous domestic cat reference genome assemblies. Assemblies are shown in chronological order with 6.2 being the earliest iteration. Loss of *DXZ4* region in subsequent assemblies is indicated by shortening of the light green arrows. Colored blocks represent stretches of contiguous sequence between the three assemblies.

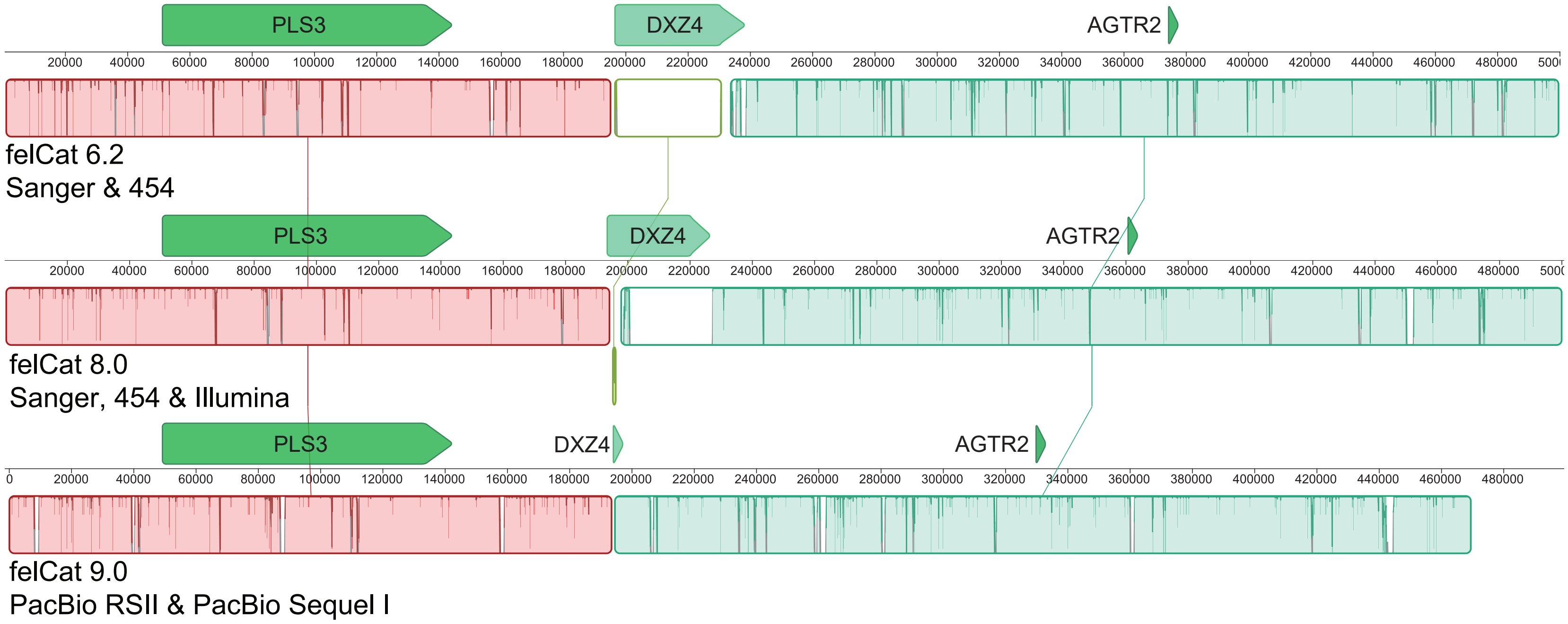

Supplementary Figure 4. Dotplots between three cat single haplotype X chromosome assemblies (y-axis) and the diploid reference, felCat9 (x-axis) reveal DXZ4 is captured within a single contig. Sequence gain was estimated from the alignment shift within each contig (y-axis), relative to the reference.

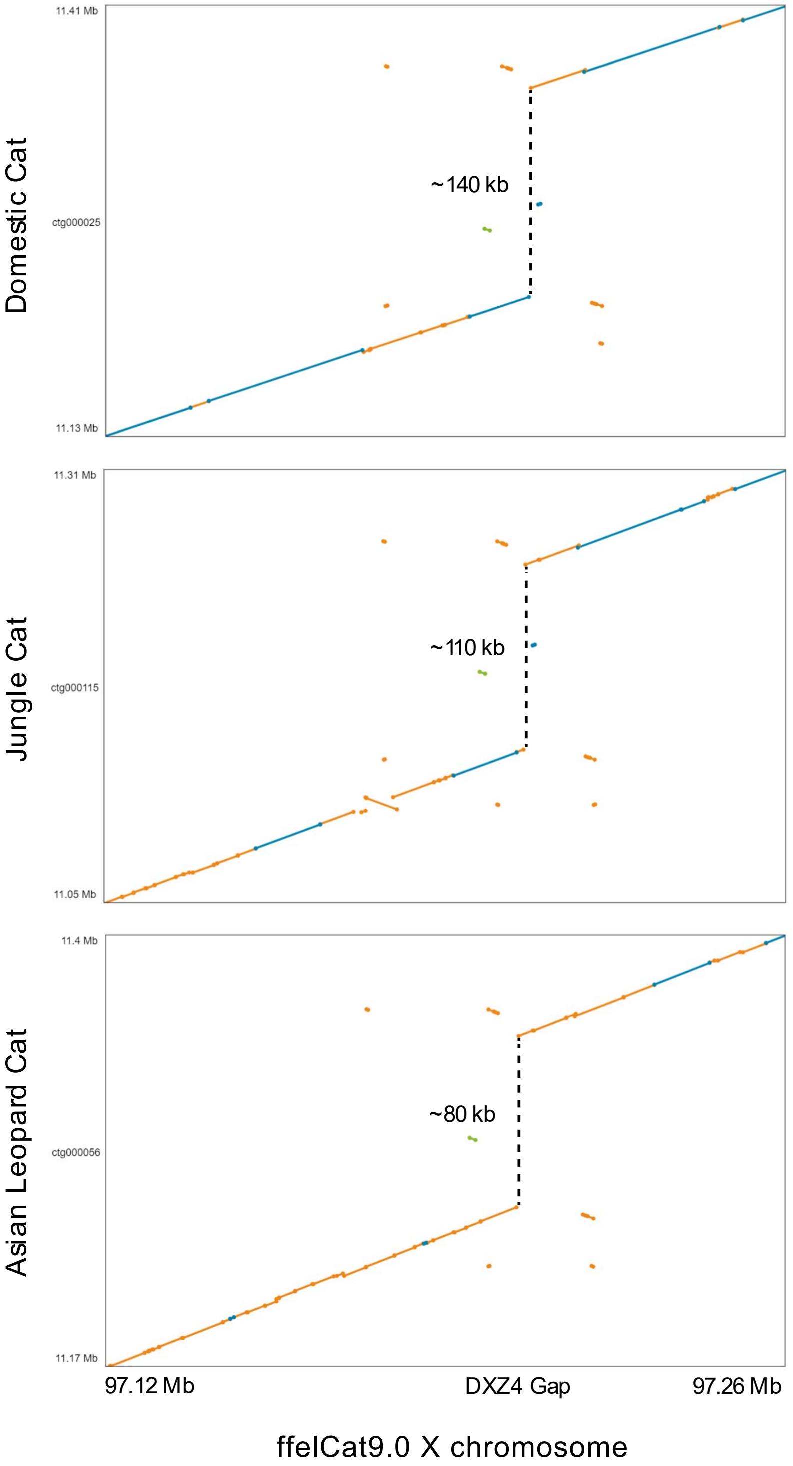

Supplementary Figure 5. Annotated *DXZ4* regions from domestic, Jungle, and Asian leopard cat assemblies.

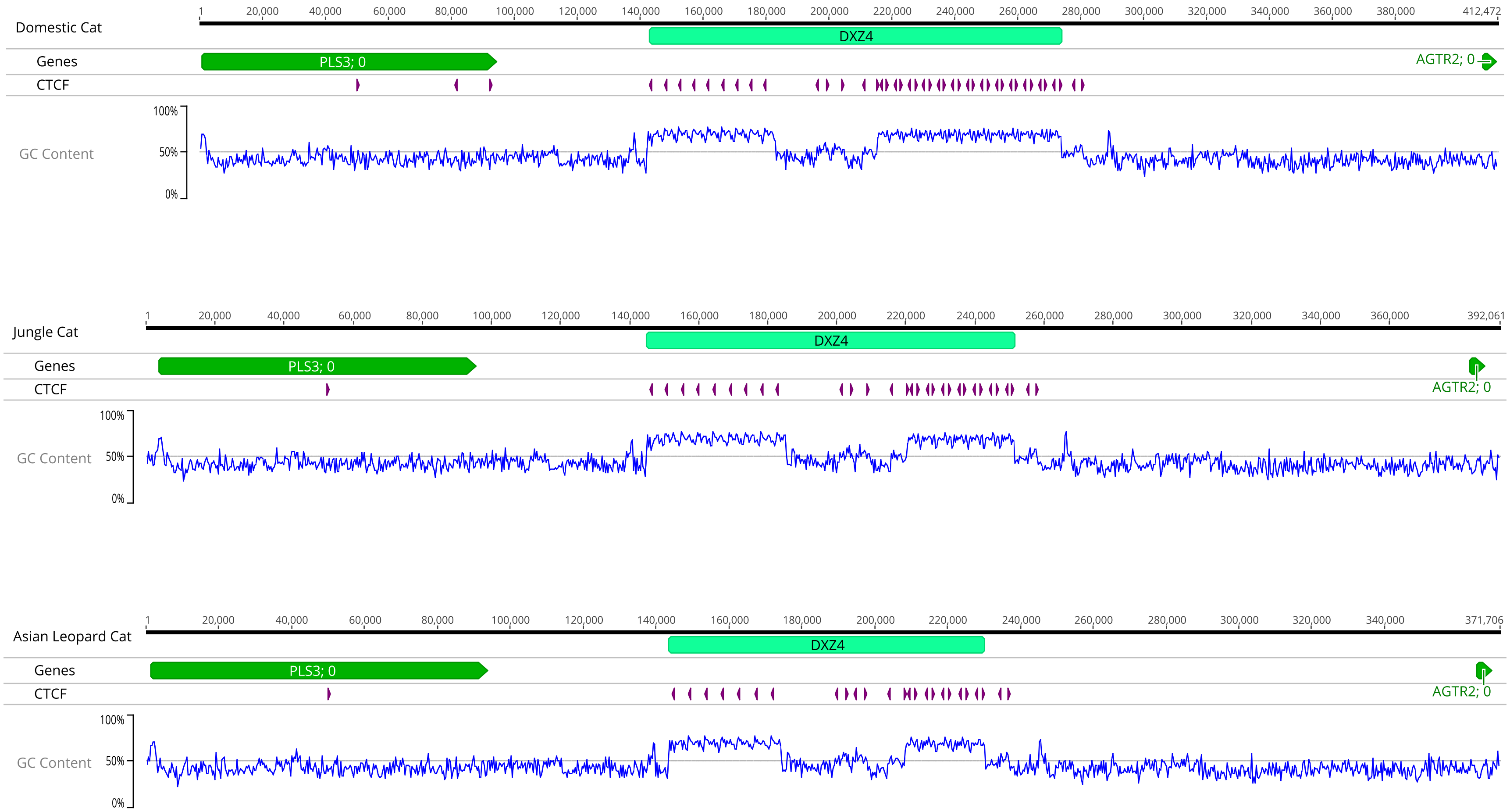

Supplementary Figure 6. *DXZ4* spacer sequence alignment. Alignment gaps distinguished by light-gray annotations.

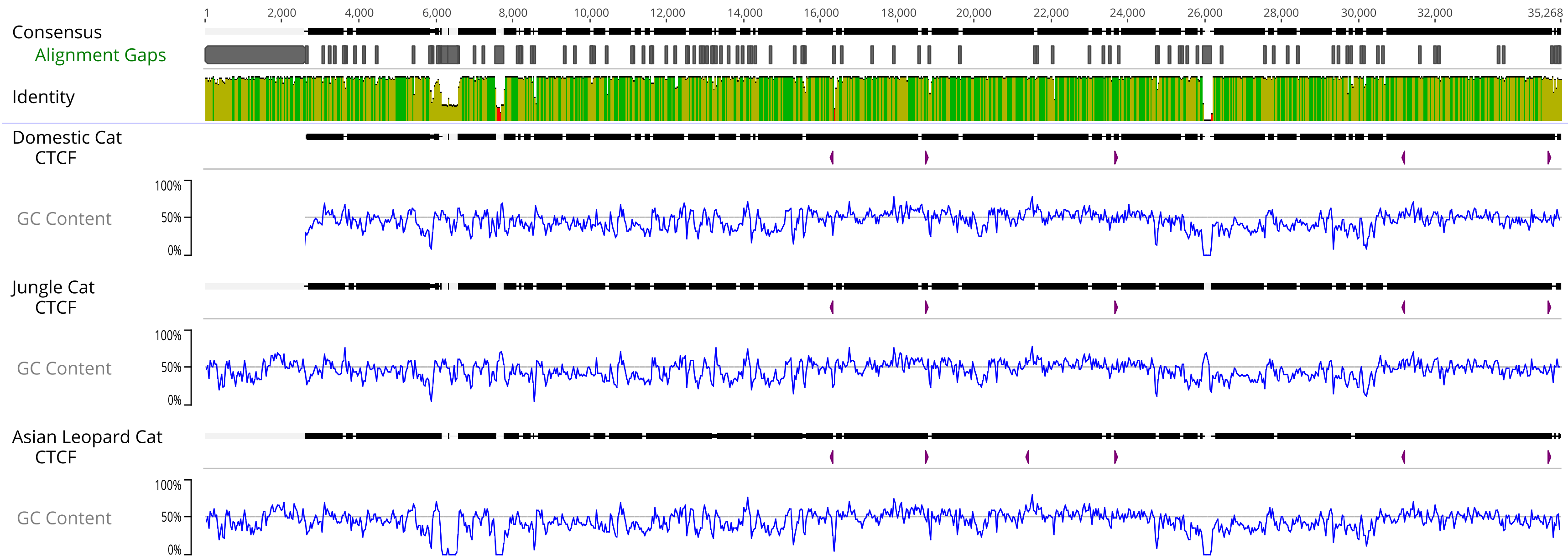

Supplementary Figure 7. *DXZ4* repeat modified for *in silico* copy number estimation using short read mapping. Unmodified *DXZ4* repeat array (Top). *DXZ4* repeat array modified for *in silico* read mapping (Bottom).

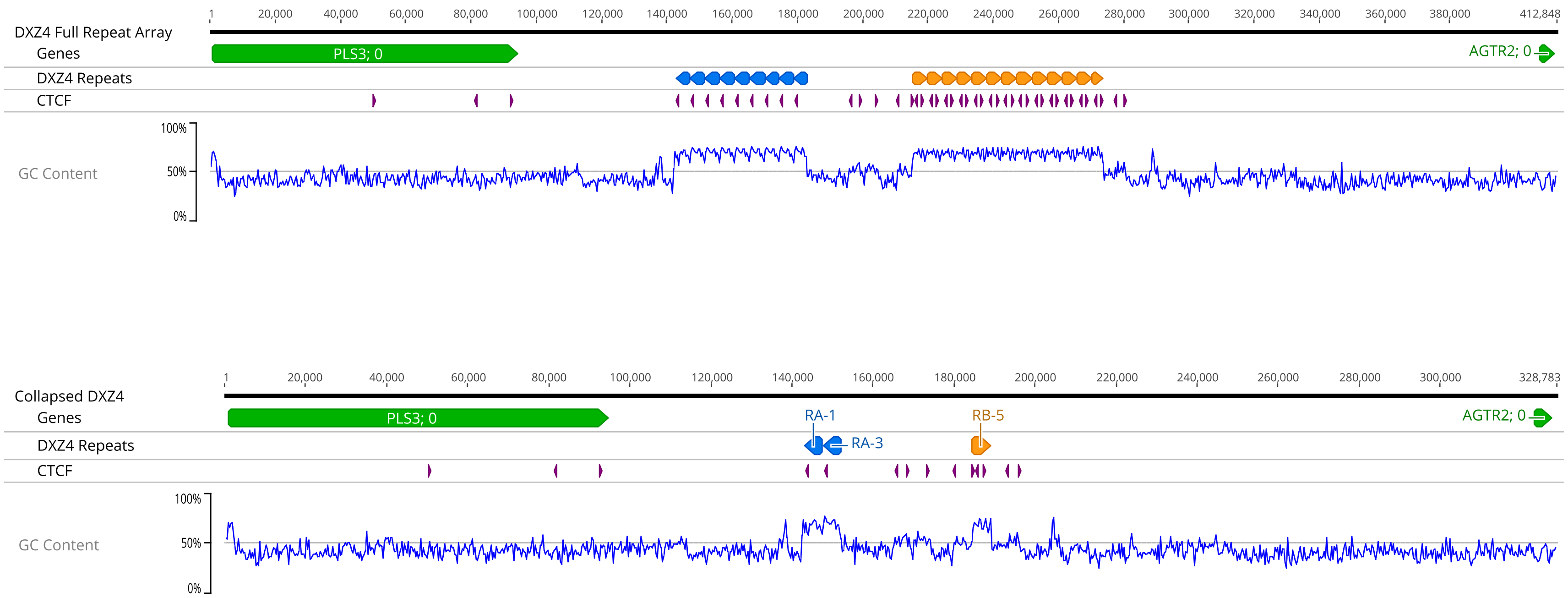

Supplementary Figure 8. Comparison between the domestic cat and human *DXZ4* annotations. Human assembly GRCh38.p13 is shown because the human T2T assembly (Miga et al, 2020) currently lacks *de novo* annotation.

Domestic cat *DXZ4* transcripts

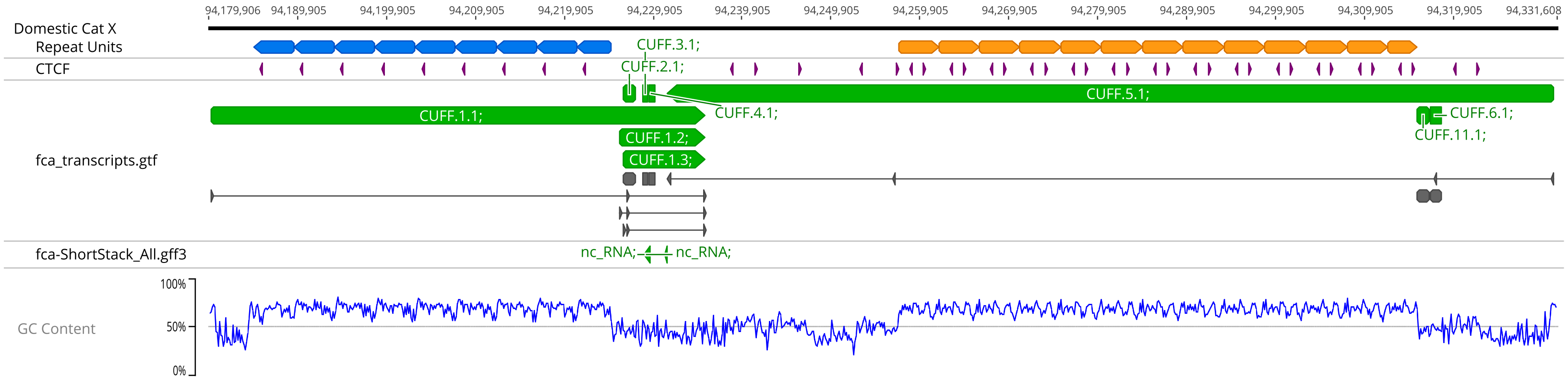

Human *DXZ4* transcripts

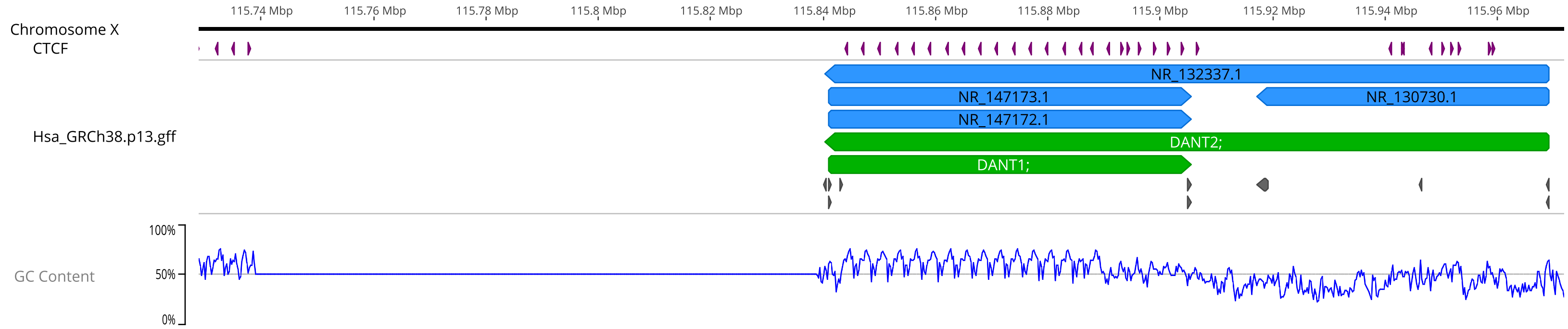

Supplementary Figure 9. Human *DANT1/2* pairwise alignments to domestic cat *CUFF1.1/5.1* (RA/RB spanning transcripts). Green bars represent regions of shared sequence identity with colored bars below reflecting different nucleotides.

Cat RA Spanning Transcript (*CUFF1.1*) vs Human *DANT1*

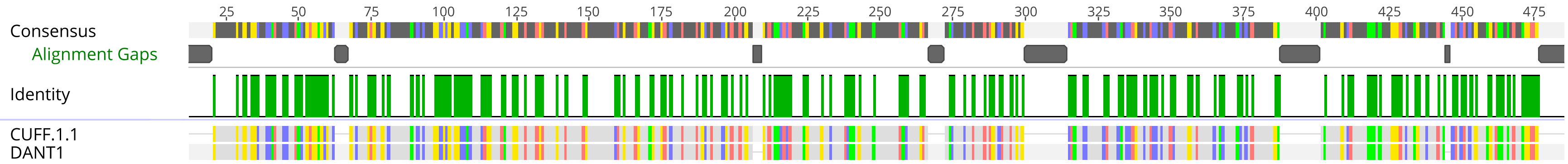

Cat RB Spanning Transcript (*CUFF5.1*) vs Human *DANT2*

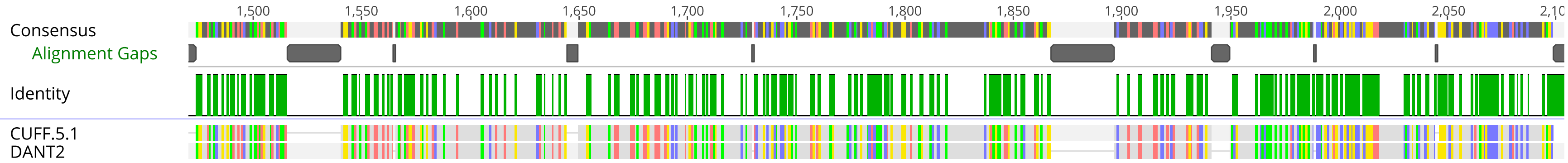

Supplementary Figure 10. Comparison between annotated *DXZ4* transcripts in domestic and Jungle cat from whole testes RNA-Seq data.

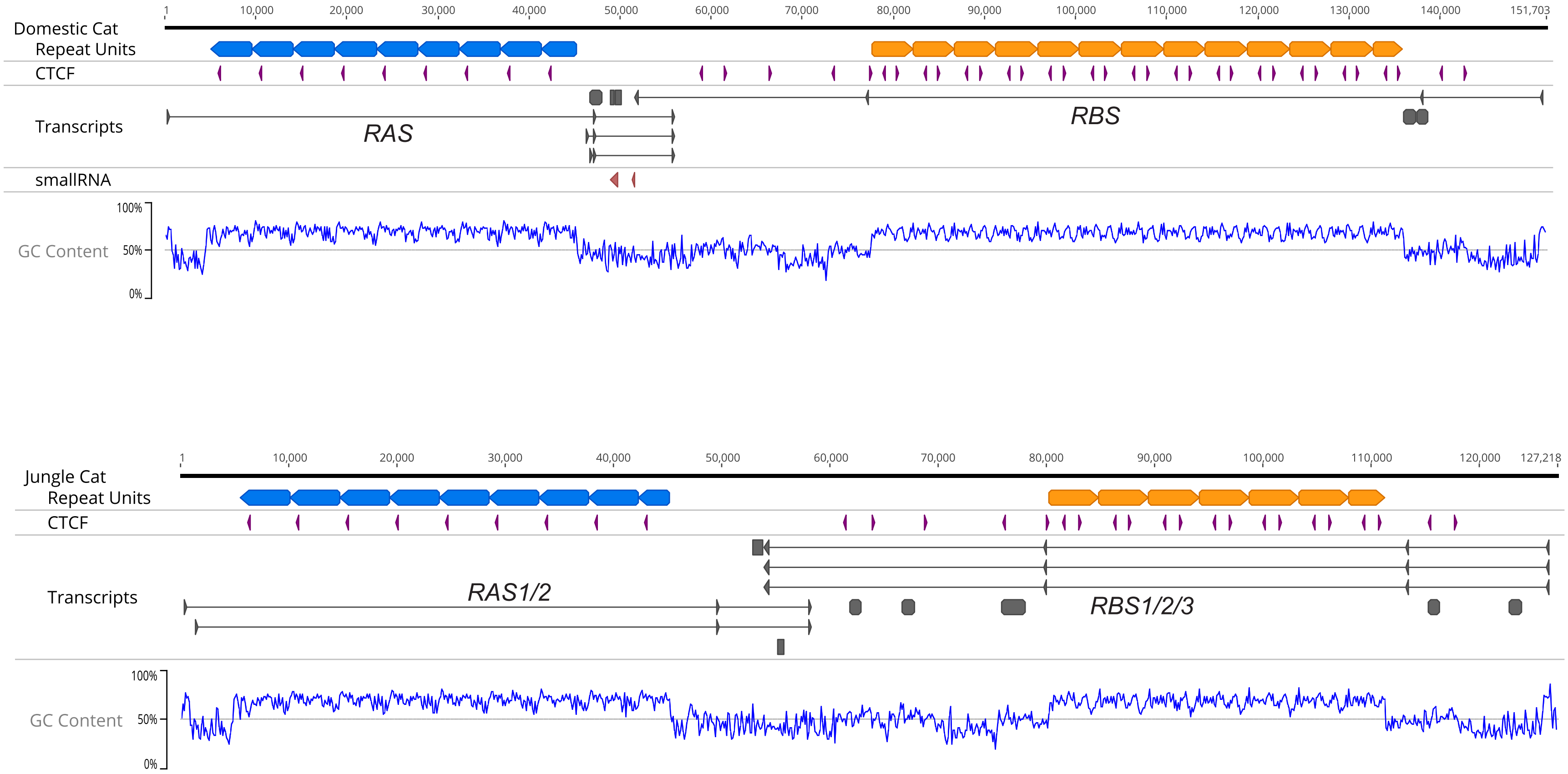

Supplementary Figure 11. Alignment between domestic and Jungle cat *DXZ4* regions. Alignment gaps are indicated by light-gray annotations.

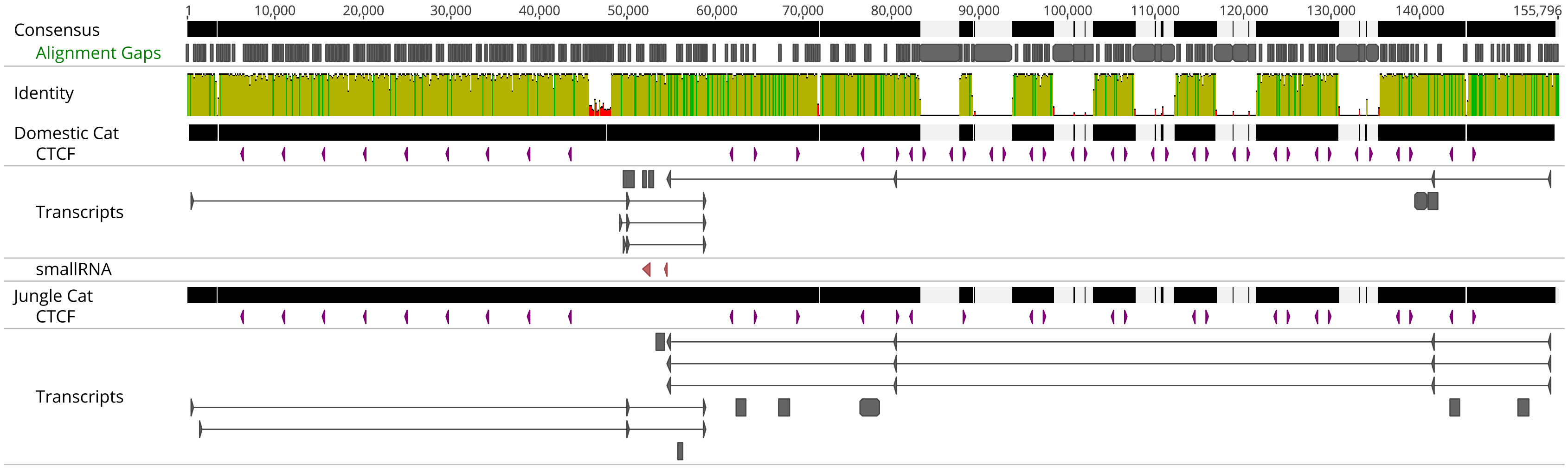

Supplementary Figure 12. allRNA and smallRNA expression across the *DXZ4* locus in domestic and Jungle cat. Y-axis scaled to 80x coverage for allRNA and 10x coverage for smallRNA in both species.

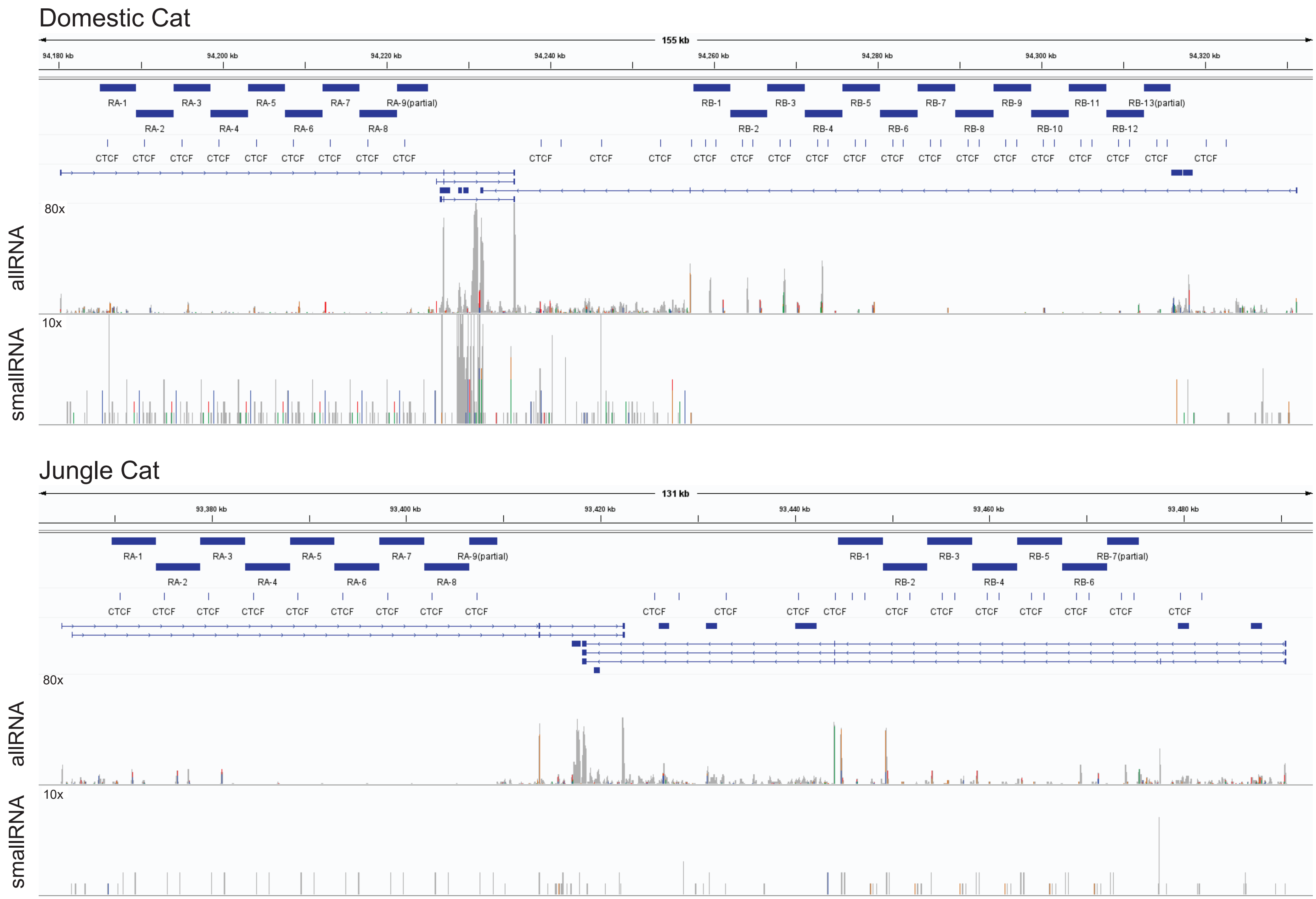

Supplementary Figure 13. Expression across first repeat unit of *DXZ4* Repeat B in both domestic and Jungle cats.

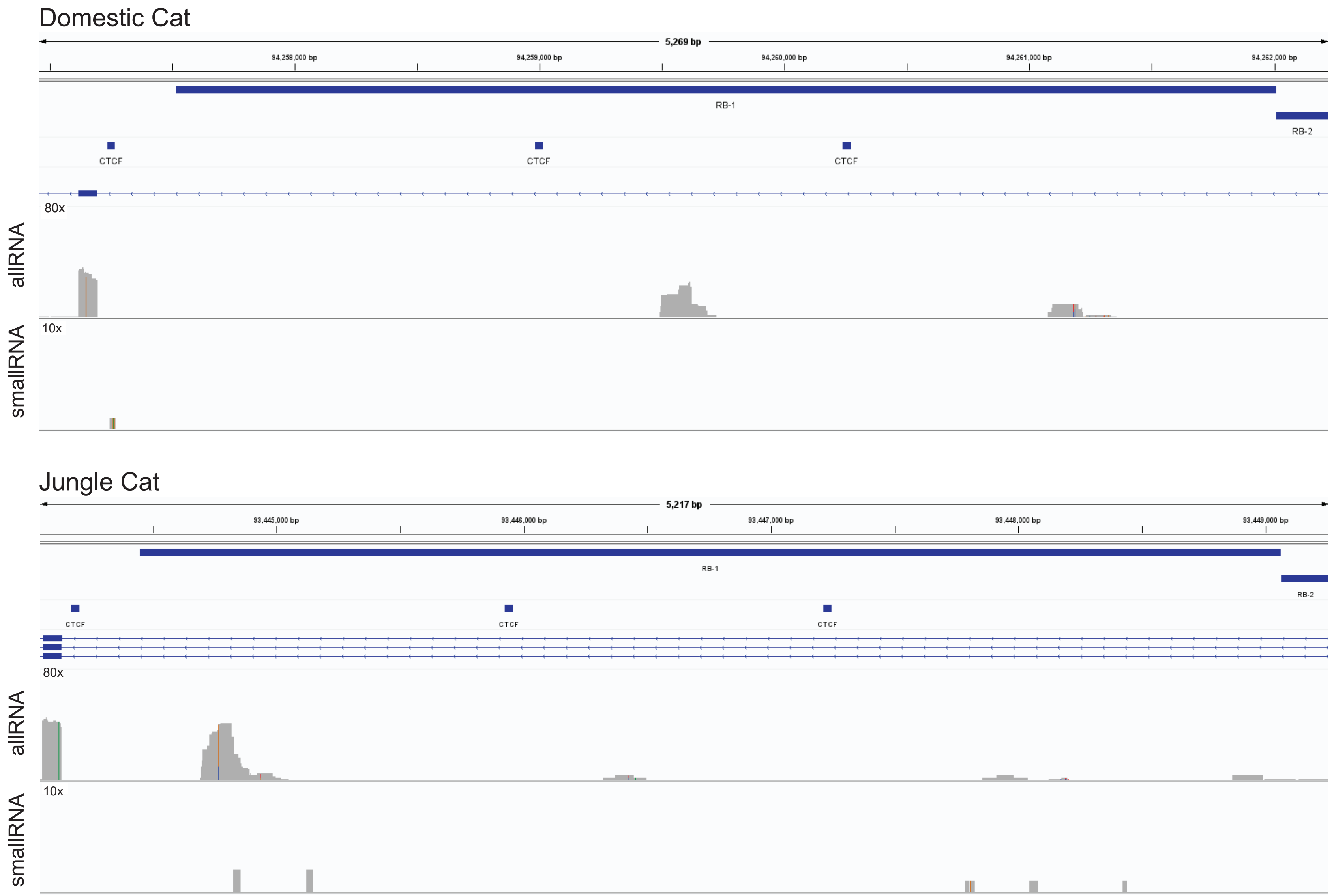

Supplementary Figure 14. RNA-seq read coverage across the *DXZ4* region for fertile and sterile Chausies. Y-axis represents raw expression scaled by highest coverage across all tracks.

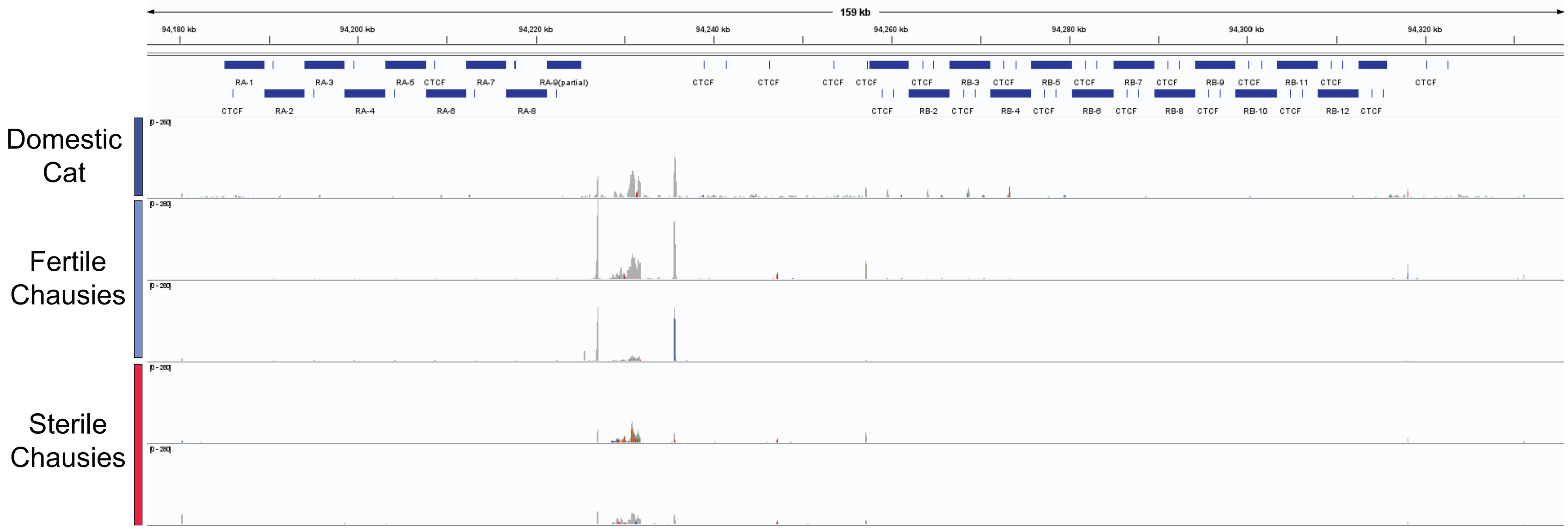

Supplementary Figure 15. PCA of methylation frequency between fertility phenotypes.

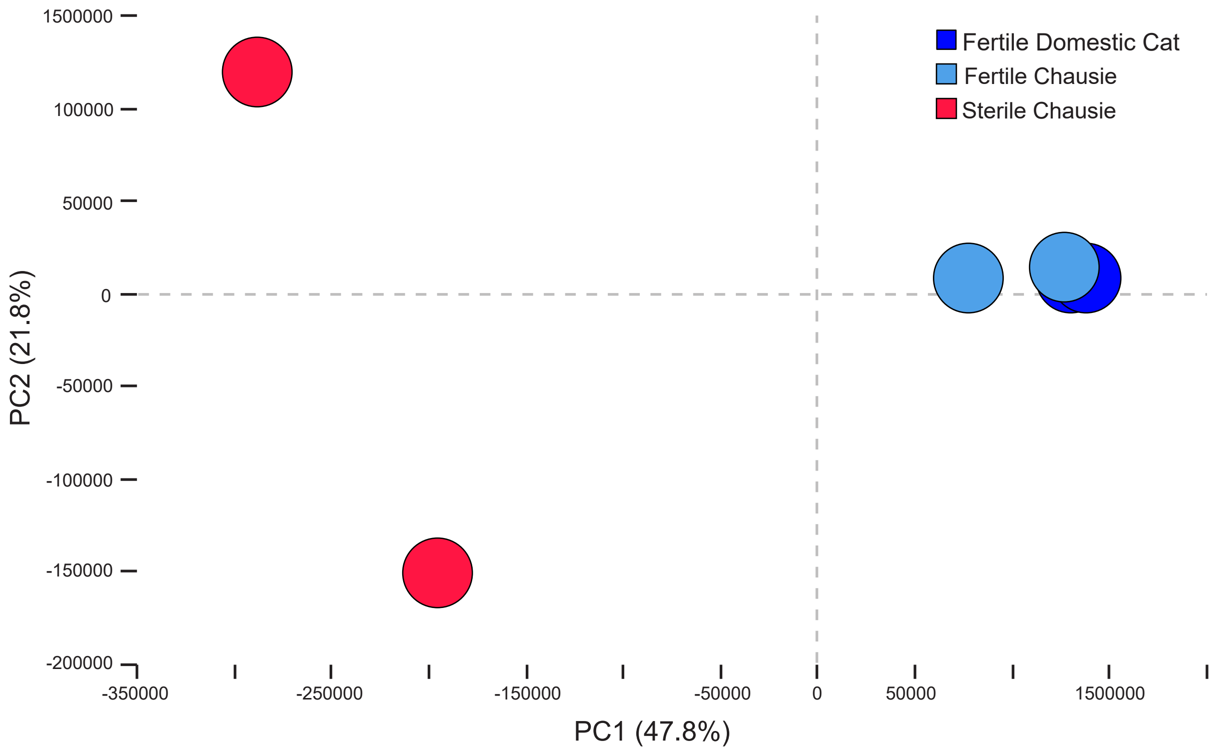

Supplementary Figure 16. Methylation frequency averages for each chromosome per each fertile or sterile individual felid. The X chromosome is indicated by an “X”.

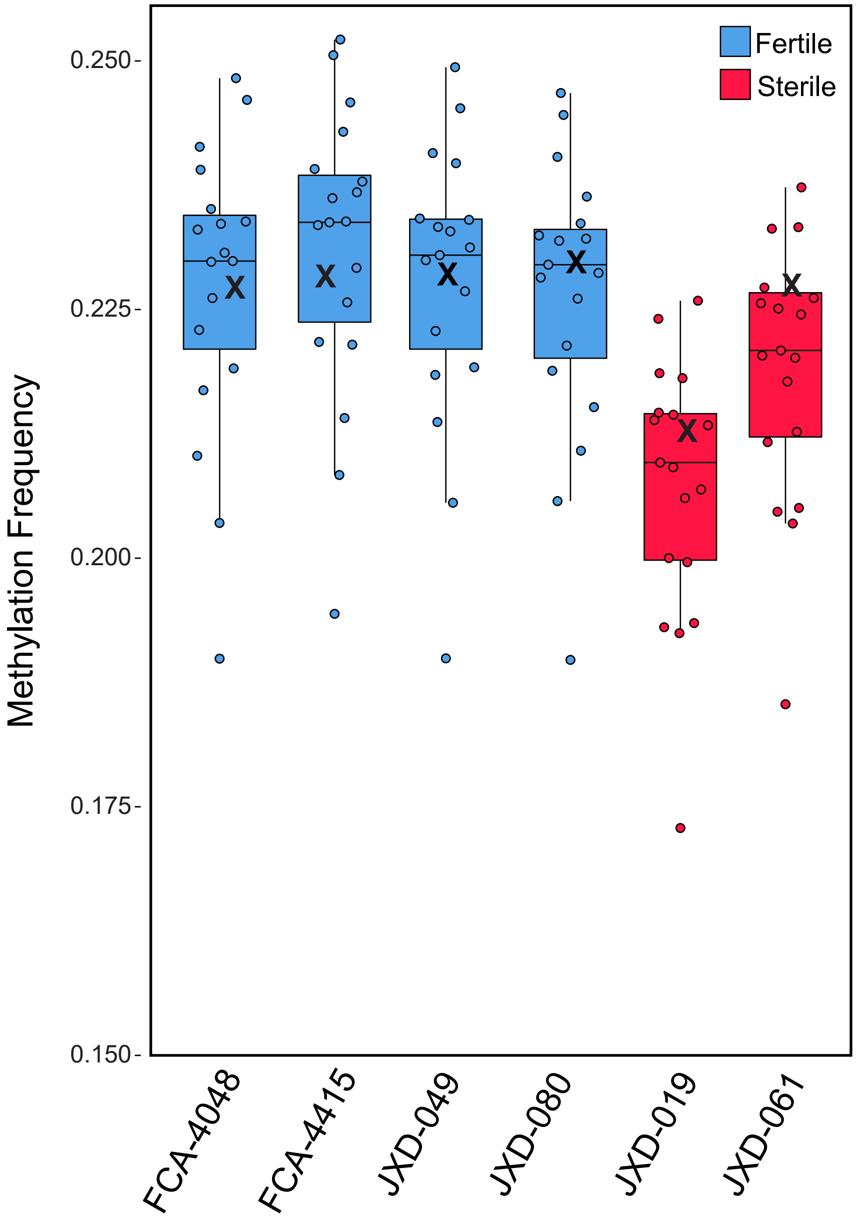

Supplementary Figure 17. Bipartite structural conformation formed by *DXZ4* is conserved on the inactive X of females in cat, human and mouse. Cat Hi-C data from phased domestic haplotype of F1 Bengal (Bredemeyer et al, 2020). Human Hi-C data from GM12878 cell line (Rao et al, 2014). Mouse Hi-C data from patski cell line (Darrow et al, 2016). Resolution is 250kb with "balanced" normalization for all maps.

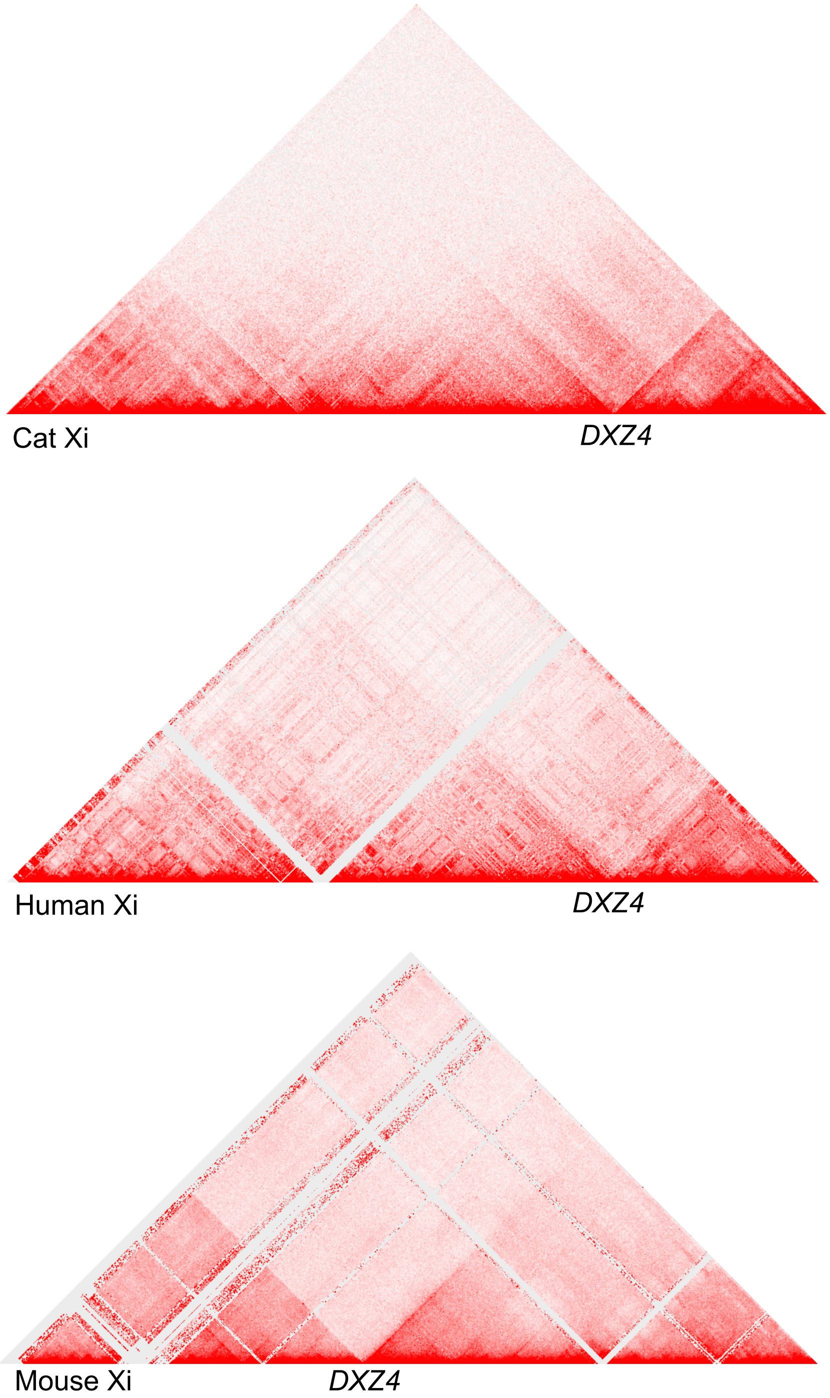

Supplementary Figure 18. Raw contact maps and Pearson's correlation maps of the X chromosome from Hi-C data generated and phased from female fibroblasts of an F1 Bengal. The domestic cat haplotype exhibits distinct features of the Xi while the Asian leopard cat haplotype resembles the Xa, suggesting potential skewing of XCI in the domestic cat haplotype.

F1 Phased Female Fibroblasts: Domestic Cat/Xi

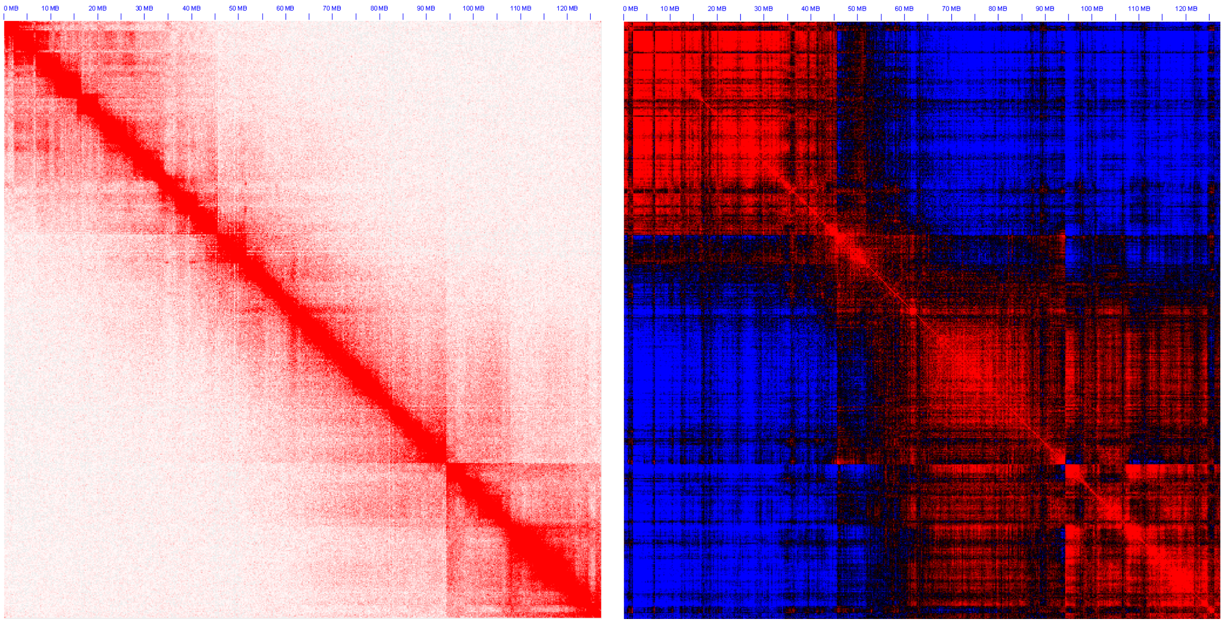

F1 Phased Female Fibroblasts: Asian Leopard Cat/Xa

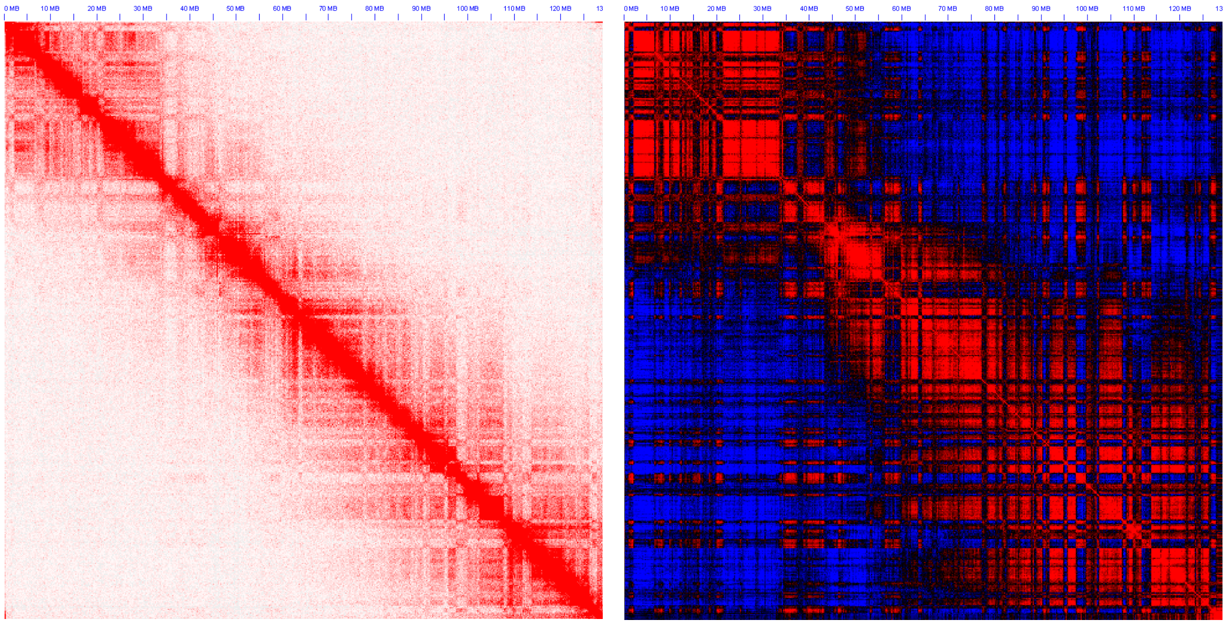

Supplementary Figure 19. Pearson's maps showing differences in compartmentalization between Xa and Xi states of cat, human (Rao et al, 2014) and mouse (Darrow et al, 2016). Cat Xa and Xi are X chromosomes from Asian leopard cat and domestic cat phased from an F1 Bengal. Resolution is 250 kb for cat and 500 kb for human and mouse.

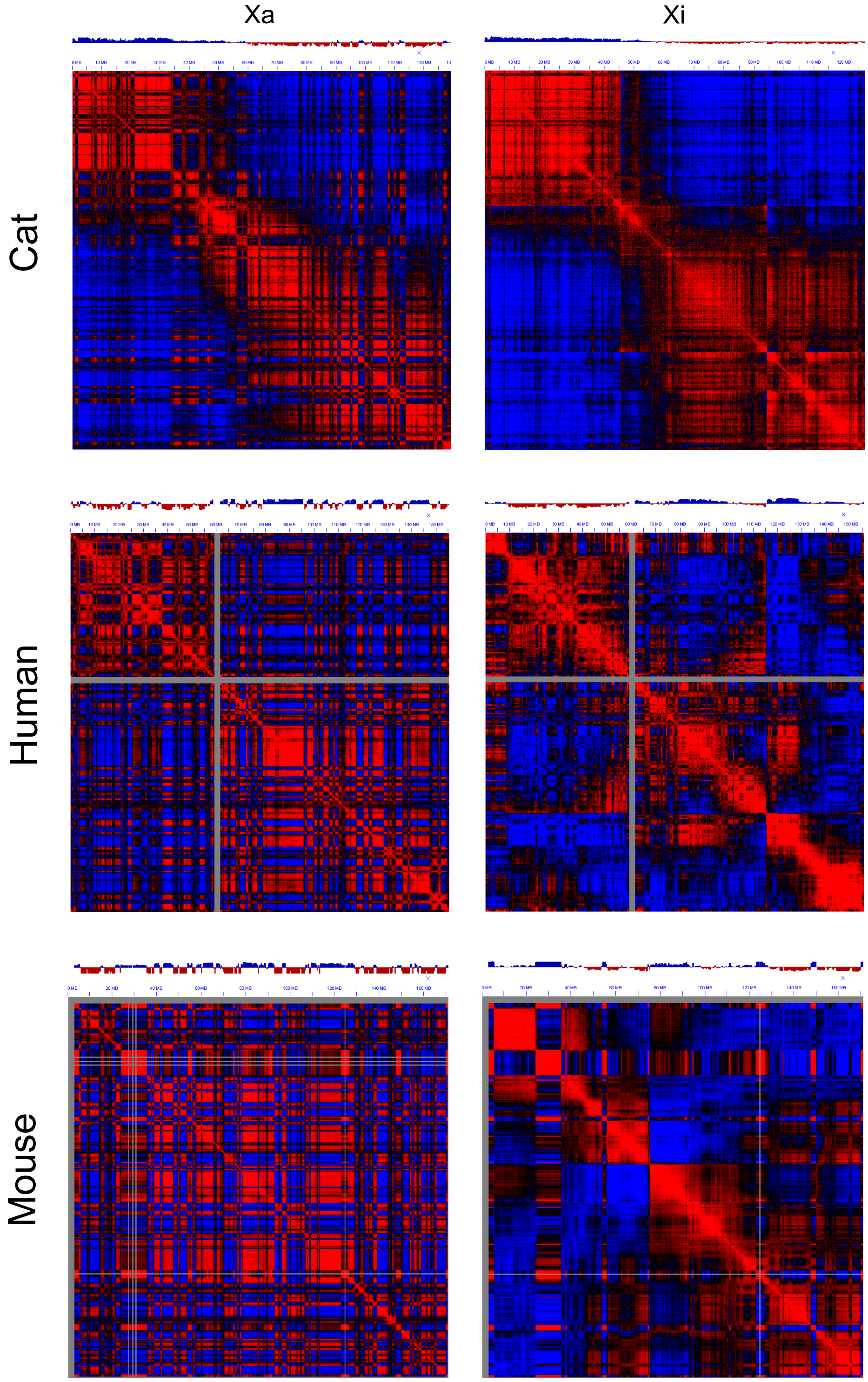

Supplementary Figure 20. *DXZ4* structural comparison between human, mouse and cat.

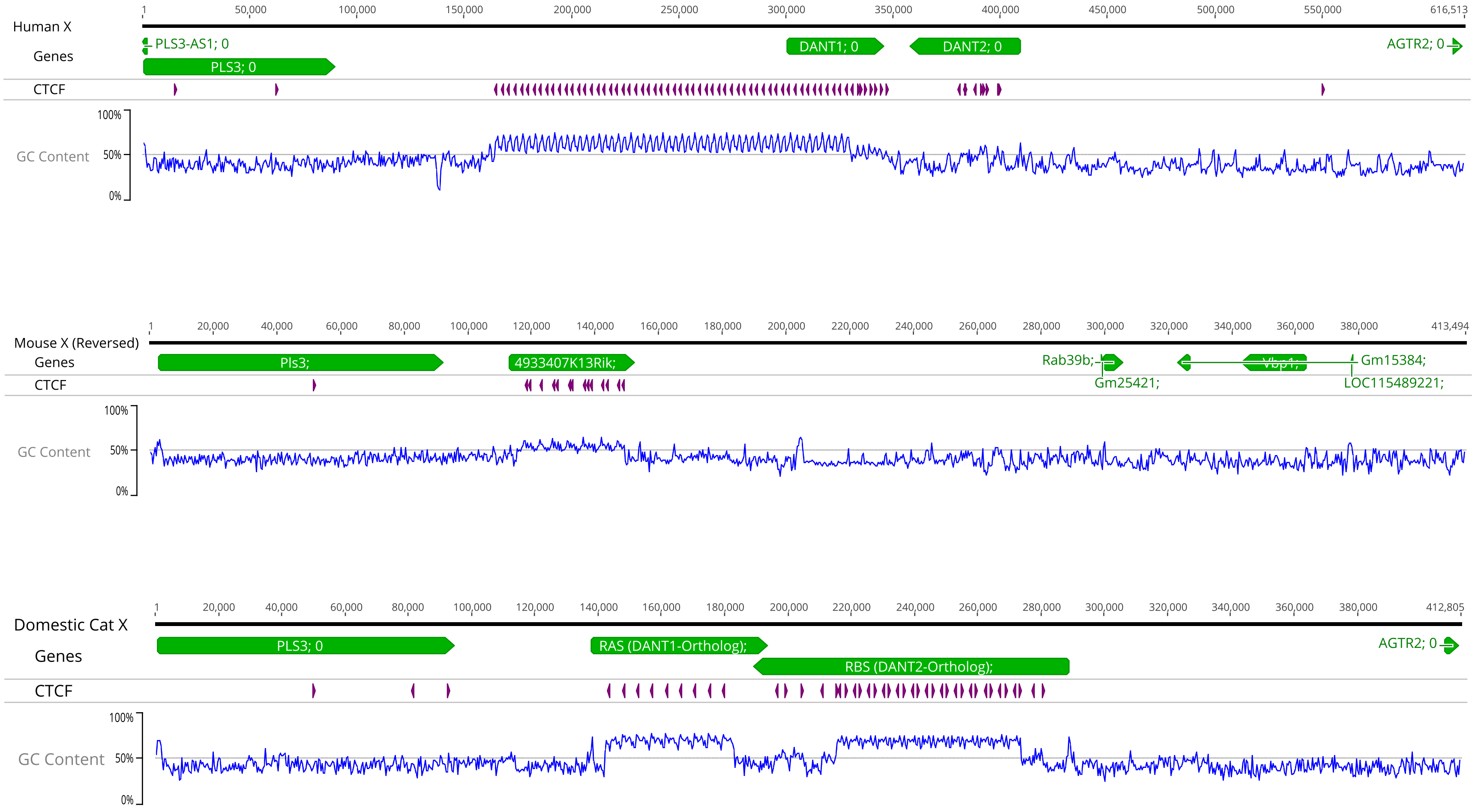

Supplementary Figure 21. Dotplot of *de novo* FelCha1.0 mitochondrial assembly and previous Jungle cat short-read mitochondrial assembly. Zoomed pane shows repetitive sequence gained in new assembly.

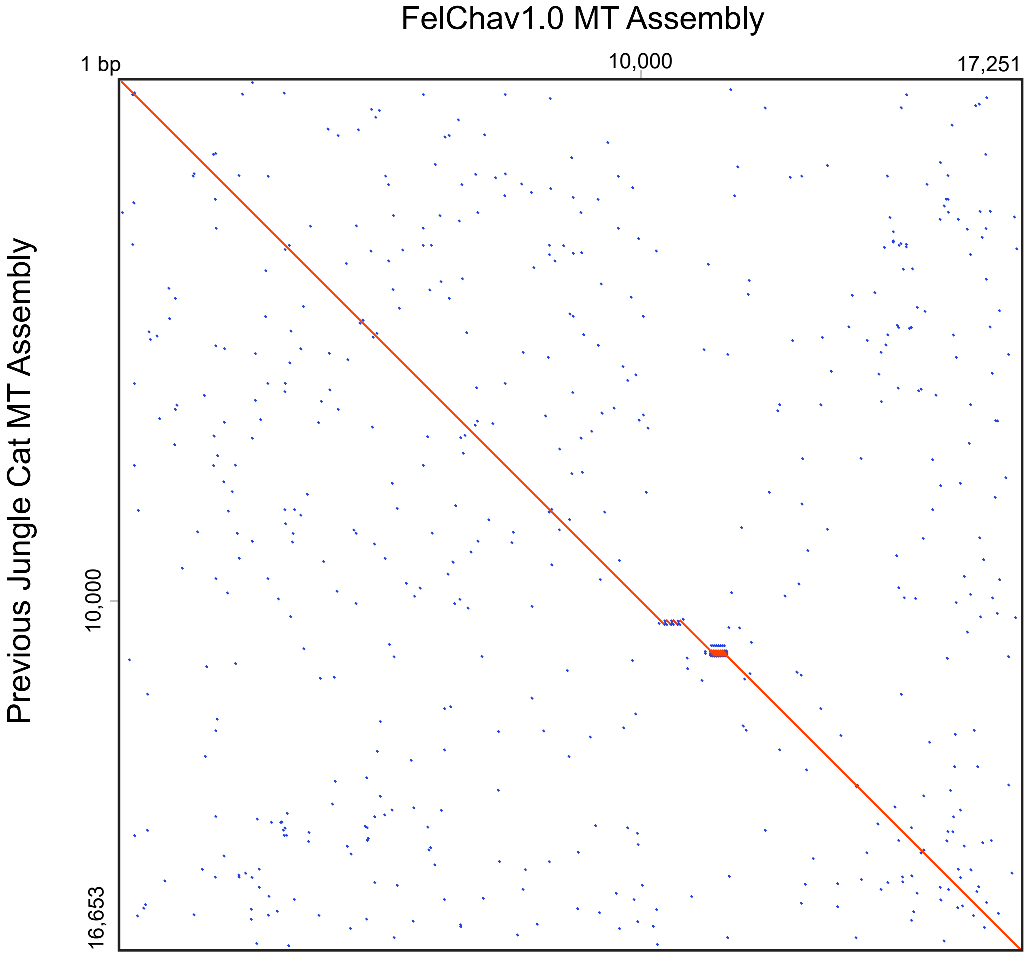

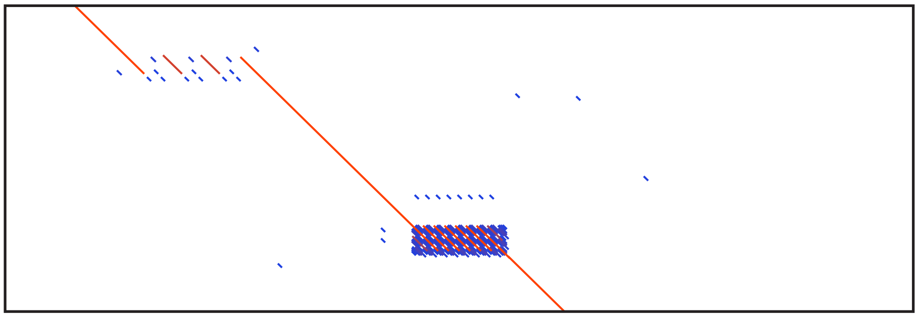

Supplementary Figure 22. Contig alignments to domestic cat single haplotype assembly (GCA_016509815.1). Chimeric contig ctg000159 representative of interchromosomal misjoin between B3 and E1 indicated by green circle.

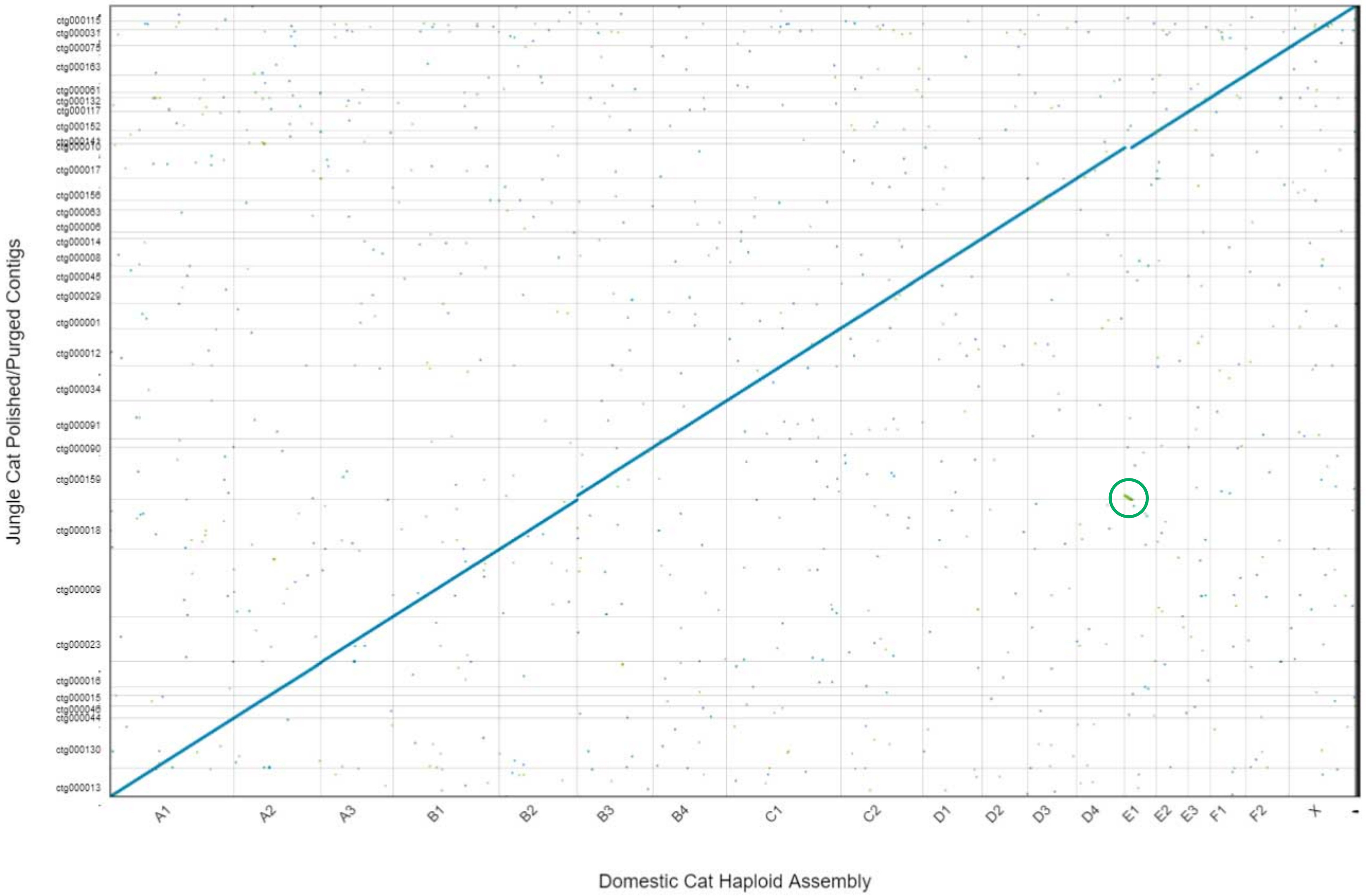

Supplementary Figure 23. Final scaffold alignments to domestic cat single haplotype assembly (GCA_016509815.1)

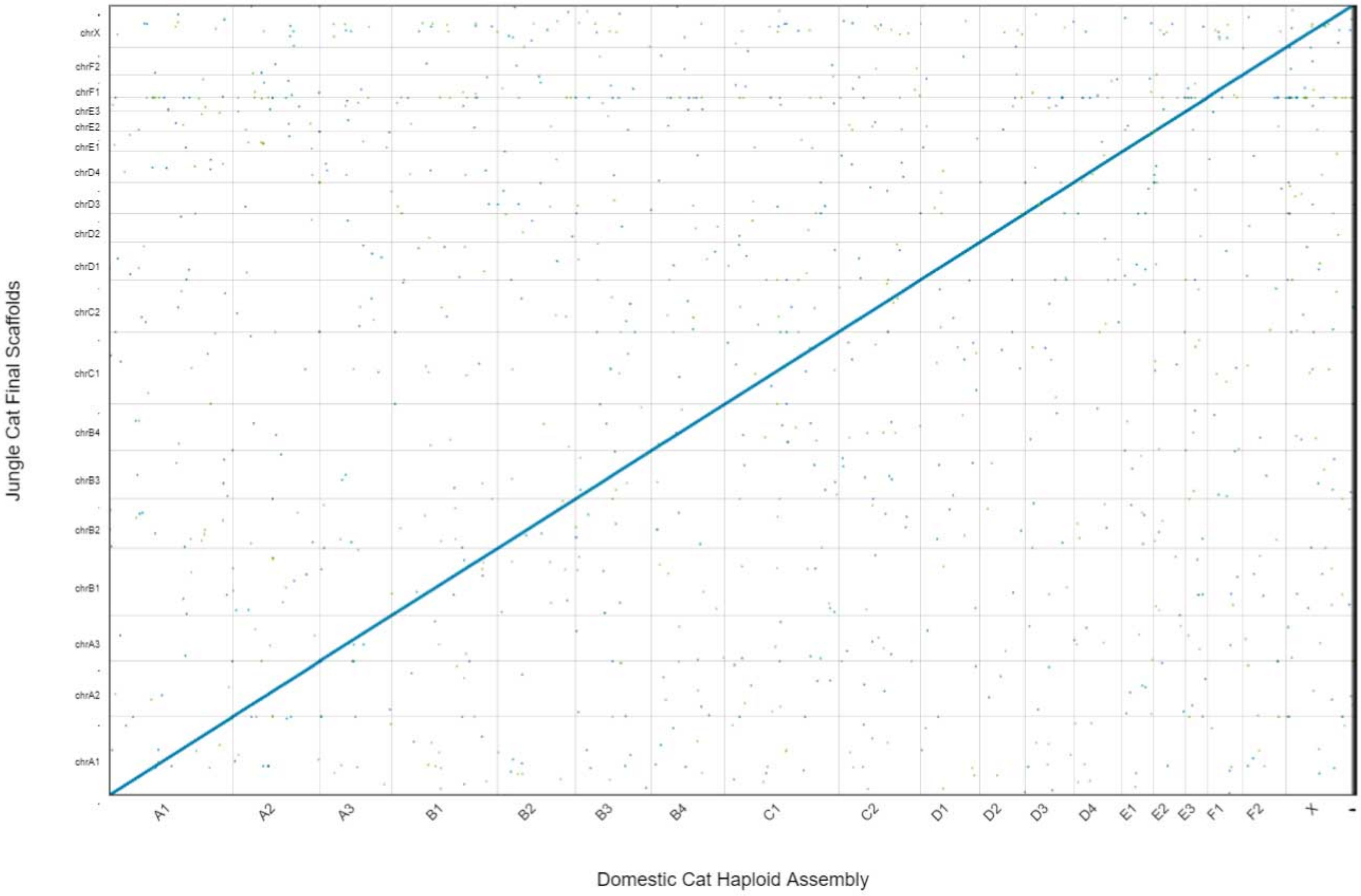

Supplementary Table 1. Gene ontology results for genes downregulated in the testis of sterile Chausie hybrids. The top ten most enriched, significant, processes involve meiotic division or spermatogenesis.

GO biological process complete	Fold Enrichment	Raw P-value	FDR
Meiotic sister chromatid segregation	7.78	1.25E-04	9.92E-03
Meiotic sister chromatid cohesion	7.78	1.25E-04	9.87E-03
piRNA metabolic process	7.41	9.00E-06	1.05E-03
Inner dynein arm assembly	7.3	2.54E-05	2.51E-03
Meiosis II	7	2.05E-04	1.46E-02
Meiosis II cell cycle process	7	2.05E-04	1.46E-02
DNA methylation involved in gamete generation	6.25	6.37E-05	5.55E-03
Epithelial cilium movement involved in extracellular fluid movement	5.71	2.16E-06	3.07E-04
Axonemal dynein complex assembly	5.63	2.45E-07	4.25E-05
Synaptonemal complex organization	5.53	2.86E-05	2.78E-03

Supplementary Table 2. Raw sequencing output by platform for the Jungle cat assembly.

Library	Reads	Bases (bp)	Genome Coverage	subread N50 (bp)	Avg. Read Length (bp)
PacBio (Sequel)	7,594,421	122,681,343,250	49x	25,928	16,154
Illumina (150bp PE)	278,636,871	83,591,061,300	33x		
Hi-C (*in situ* DNase)	1,541,076,320	462,322,896,000	185x		

Supplementary Table 3. Interspecific *P*-distance for *DXZ4* repeat A (RA) units with and without outlier RA-1, spacer sequence and repeat B (RB) units. *P*-distance was calculated for shared alignment sites only. Results indicate increased divergence across both RA and RB relative to the spacer sequence.

Interspecific Mean *P*-Distance

RA

	Domestic	Jungle
Domestic Cat		
Jungle Cat	0.0276	
Asian Leopard Cat	0.0401	0.0429

RA excluding RA-1

	Domestic	Jungle
Domestic Cat		
Jungle Cat	0.0165	
Asian Leopard Cat	0.0296	0.0320

Spacer

	Domestic	Jungle
Domestic Cat		
Jungle Cat	0.0103	
Asian Leopard Cat	0.0181	0.0176

RB

	Domestic	Jungle
Domestic Cat		
Jungle Cat	0.0203	
Asian Leopard Cat	0.0266	0.0304

Supplementary Table 4. Repeat unit length summary for *DXZ4* tandem arrays from the Jungle cat *de novo*, domestic and Asian leopard cat single haplotype assemblies.

Species	Repeat A	Length (bp)
Fca	RA-1	4,481
Fca	RA-2	4,544
Fca	RA-3	4,544
Fca	RA-4	4,544
Fca	RA-5	4,544
Fca	RA-6	4,576
Fca	RA-7	4,544
Fca	RA-8	4,512
**Fca Average (no RA-1)**		**4,544**
Fch	RA-1	4,529
Fch	RA-2	4,576
Fch	RA-3	4,624
Fch	RA-4	4,576
Fch	RA-5	4,608
Fch	RA-6	4,608
Fch	RA-7	4,608
Fch	RA-8	4,608
**Fch Average (no RA-1)**		**4,601**
Pbe	RA-1	4,545
Pbe	RA-2	4,512
Pbe	RA-3	4,544
Pbe	RA-4	4,512
Pbe	RA-5	4,512
Pbe	RA-6	4,544
**Pbe Average (no RA-1)**		**4,525**
**RA Average**		**4,554**
**RA StDev**		**39**

Species	Repeat B	Length (bp)
Fca	RB-1	4,494
Fca	RB-2	4,554
Fca	RB-3	4,584
Fca	RB-4	4,584
Fca	RB-5	4,615
Fca	RB-6	4,584
Fca	RB-7	4,615
Fca	RB-8	4,615
Fca	RB-9	4,584
Fca	RB-10	4,614
Fca	RB-11	4,615
Fca	RB-12	4,615
**Fca Average**		**4,589**
Fch	RB-1	4,616
Fch	RB-2	4,590
Fch	RB-3	4,640
Fch	RB-4	4,589
Fch	RB-5	4,615
Fch	RB-6	4,615
**Fch Average**		**4,611**
Pbe	RB-1	4,673
Pbe	RB-2	4,630
Pbe	RB-3	4,651
Pbe	RB-4	4,651
**Pbe Average**		**4,651**
**RB Average**		**4,607**
**RB StDev**		**36**

Supplementary Table 5. *DXZ4 in silico* copy number estimates.

	Breed	RA	RB	Total	Genome Coverage
**Domestic Cats**					
Fca-508 SHA*	Assembly	9	13	22	-
Iraq	Domestic Shorthair Outbred	28	9	37	26x
Mateo	Domestic Shorthair Outbred	12	12	24	35x
Danny Boy	Domestic Shorthair Outbred	9	13	22	36x
Portugal	Domestic Shorthair Outbred	7	7	14	24x
Sizzle	Domestic Shorthair Outbred	4	7	11	23x
Thailand	Domestic Shorthair Outbred	2	5	7	26x
Loki	Domestic Shorthair Outbred	1	4	5	50x
**Outbred Average**		**9**	**8**	**17**	**-**
**Outbred SD**		**8.6**	**3.1**	**10.4**	**-**
Rocket	Maine Coon	7	9	16	29x
Tennessee	Tennessee Rex	4	8	12	29x
Speckles	Peterbald	4	6	10	35x
Gannon	Egyptian Mau	3	8	11	28x
Marcus	Persian	2	7	9	36x
**Breed Average**		**4**	**8**	**12**	**-**
**Breed SD**		**1.7**	**1.0**	**2.4**	**-**
**Domestic Average**		**7**	**8**	**15**	**31x**
**Domestic SD**		**6.8**	**2.8**	**8.5**	**7.1x**
**Jungle Cats**					
Fch-1a (FelCha1.0)	Assembly	9	7	16	-
Fch-12		5	3	8	18x
**Average**		**7**	**5**	**12**	**18x**
**Asian Leopard Cats**					
Pbe-53 SHA*	Assembly	7	5	12	-
Pbe-38		6	3	9	13x
**Average**		**7**	**4**	**11**	**13x**

*Single Haplotype Assembly

Supplementary Table 6. Differential methylation of *DXZ4* across 8 felid testis or sorted germ cell samples. (Abbreviations: MF, methylation frequency)

**Phenotype**	**Felid group**	**Sample ID**	*DXZ4* gene **Mean MF**
Fertile	Domestic cat	Pachytene	0.181
	Domestic cat	Spermatid	0.151
	Domestic cat	FCA-4048	0.260
	Domestic cat	FCA-4415	0.268
	Chausie	JXD-049	0.246
	Chausie	JXD-080	0.262
Sterile	Chausie	JXD-019	0.302
	Chausie	JXD-061	0.322

Supplementary Table 7. Windows with significant differential methylation of testes in *DXZ4* Repeat Array A on chromosome X between fertile (n, domestic cat=2, Chausie=2) and sterile (n, Chausie=2) felids. Significance was determined by 1-tailed t-test of unequal variance.

Window start	Window stop	*p-value*
94,185,541	94,185,607	0.0388
94,185,567	94,185,797	0.0485
94,185,601	94,185,817	0.0054
94,185,788	94,185,829	2.95×10^{-7}
94,185,816	94,185,839	7.30×10^{-5}
94,185,828	94,185,851	1.25×10^{-3}
94,185,838	94,185,866	6.26×10^{-4}
94,185,849	94,185,903	9.84×10^{-4}
94,185,865	94,185,952	5.22×10^{-3}
94,185,900	94,186,070	0.0231
94,185,939	94,186,117	7.23×10^{-3}
94,186,069	94,186,173	0.0133
94,186,114	94,186,200	9.92×10^{-3}
94,186,171	94,186,225	3.51×10^{-3}
94,186,199	94,186,399	3.92×10^{-3}
94,186,224	94,186,437	0.0297
94,186,423	94,186,481	0.0472
94,186,453	94,186,506	0.0211
94,186,923	94,186,975	0.0480
94,186,943	94,187,001	0.0134
94,186,972	94,187,487	0.0106
94,186,998	94,187,500	0.0210
94,187,910	94,188,198	0.0368
94,188,176	94,188,233	0.0485
94,188,297	94,188,653	1.33×10^{-3}
94,188,316	94,188,762	3.77×10^{-5}
94,188,651	94,188,790	1.02×10^{-3}
94,189,268	94,189,317	0.0422
94,189,291	94,189,317	0.0176

Supplementary Table 8. SRA accessions of individuals used for *in silico DXZ4* copy number estimations.

Species	Identifier	SRA	Sex	Ancestry
Domestic Cat	Sizzle	SRR5055407	Male	Established Breed
Domestic Cat	Loki	SRR5055386	Male	Established Breed
Domestic Cat	Rocket	SRR5051106	Male	Established Breed
Domestic Cat	Gannon	SRR5051108	Male	Established Breed
Domestic Cat	Marcus	SRR2224864	Male	Established Breed
Domestic Cat	Flowmaster	SRR5051112	Male	Established Breed
Domestic Cat	TennesseeTom	SRR5051114	Male	Established Breed
Domestic Cat	Speckles	SRR5051122	Male	Established Breed
Domestic Cat	Fca-508	SRR12914279	Female	Domestic Shorthair Outbred
Domestic Cat	Mateo	SRR11392568	Male	Domestic Shorthair Outbred
Domestic Cat	DannyBoy	SRR11392571	Male	Domestic Shorthair Outbred
Domestic Cat	Portugal-350<br>Portugal-550	SRR5040114<br>SRR5040108	Male	Domestic Shorthair Outbred
Domestic Cat	Iraq-350<br>Iraq-550	SRR5040113<br>SRR5040123	Male	Domestic Shorthair Outbred
Domestic Cat	Thailand-350<br>Thailand-550	SRR5040120<br>SRR5040112	Male	Domestic Shorthair Outbred
Jungle Cat	Fch-1a	SRR13340505	Male	
Jungle Cat	Fch-12	SRR2062187	Female	
Asian Leopard Cat	Pbe-53	SRR12914278	Male	
Asian Leopard Cat	Pbe-38	SRR4426179	Female	

Supplementary Table 9. Meta-data for each of the felid testis samples included in this study.

Sample ID	Phenotype	Age at neuter (years)	Breeder estimated Jungle cat ancestry
Whole testis tissue			
FCA-4415	Fertile Domestic Cat	3	
FCA-4048	Fertile Domestic Cat	2.5	
JXD-019	Sterile Chausie	1.5	14%
JXD-049	Fertile Chausie	1.75	13%
JXD-061	Sterile Chausie	2	14%
JXD-080	Fertile Chausie	3	14%
Germ cells			
Pachytene	Fertile Domestic Cat	1.5	
Spermatid	Fertile Domestic Cat	1.5	

Supplementary Table 10. Number of RRBS raw and uniquely mapped reads, percent mappability (M) to the reference genome, bisulfite conversion (BSconv) proportions, percent of cytosines within each methylation motif (mCG, mCHG, mCHH), and methylation frequency (MF) across all chromosomes.

Sample ID	Batch	Raw reads	Uniquely aligned reads	M	BSconv	mCG	mCHG	mCHH	MF*
FCA-4415	1	34,135,894	28,227,690	82.7	0.94	77.7	11	9.8	0.231
FCA-4048	1	33,582,352	27,711,201	82.5	0.94	78.1	9.8	8.7	0.228
JXD-019	1	38,683,216	30,015,515	77.6	0.95	72.3	8.8	7.9	0.207
JXD-049	1	34,555,044	27,925,207	80.8	0.94	77	12.4	11.3	0.228
JXD-061	1	32,703,912	24,615,157	75.3	0.94	74.9	11.9	10.9	0.219
JXD-080	1	31,531,246	25,114,703	79.7	0.94	74.9	11	9.8	0.227
Pachytene	2	27,264,057	16,041,684	58.8	0.97	64.8	5.1	4.3	--
Spermatids	2	28,818,849	16,917,143	58.7	0.97	59.6	3.1	2.6	--

*After filtering sites for 10x coverage and uniting all testes data

Supplementary Table 11. Y-linked contigs identified using BLAST. Ctg000067-2 is the Y specific region originally included in ctg000067, which composed the pseudoautosomal region. ctg000067-2, ctg000078, and ctg000135 all contained genes belonging to the single copy region of the Y chromosome. These were manually scaffolded and identified in the final assembly as chrY. Annotations were mapped to each contig using liftover from the domestic cat published Y chromosome sequence.

Contig ID	Length (bp)	Annotations
ctg000067-2	206,618	*FLJ36031Y-d340, FLJ36031Y-e268*
ctg000078	564,111	*TETY2, UTY, DDX3Y, USP9Y*
ctg000135	482,115	*AMELY, EIF2S3Y, ZFY, EIF1AY, RPS4Y-A, RPS4Y-B, RPS4Y-C*
ctg000072	409,643	*LOC109496917, LOC111561459*
ctg000074	72,713	*TSPY-b274*
ctg000077	317,206	*TSPY-a244, TSPY-c264, Cyorf_fusion, SRY*
ctg000100	214,609	None
ctg000101	326,606	*FLJ36031Y-a260, FLJ36031Y-b321, FLJ36031Y-c254, FLJ36031Y-d340, FLJ36031Y-e268*
ctg000128	63,486	*TETY1*
ctg000136	229,506	*RPS4Y-A, RPS4Y-B, RPS4Y-C, HSFY*
ctg000137	214,959	*RPS4Y-A, RPS4Y-C, HSFY*
ctg000138	48,132	*RPS4Y-A, RPS4Y-B, RPS4Y-C, HSFY*
ctg000150	69,303	*Cyorf_orig*
ctg000171	256,672	*FLJ36031Y-d340, FLJ36031Y-e268*
**Total Length**	**3,475,679**	

Supplementary Table 12. Jungle cat genome assembly (FelCha1.0).

Molecule	Total Length (bp)	Gaps	Ungapped Length (bp)
**ALL**	**2,428,287,114**	**57**	**2,428,281,414**
Chromosome A1	240,008,610	2	240,008,410
Chromosome A2	169,335,317	8	169,334,517
Chromosome A3	140,691,898	1	140,691,798
Chromosome B1	205,710,267	0	205,710,267
Chromosome B2	152,756,071	0	152,756,071
Chromosome B3	148,552,997	1	148,552,897
Chromosome B4	142,338,932	1	142,338,832
Chromosome C1	222,028,171	4	222,027,771
Chromosome C2	159,171,038	1	159,170,938
Chromosome D1	115,689,139	2	115,688,939
Chromosome D2	88,529,372	2	88,529,172
Chromosome D3	95,225,590	2	95,225,390
Chromosome D4	95,074,675	4	95,074,275
Chromosome E1	61,214,427	3	61,214,127
Chromosome E2	61,992,405	1	61,992,305
Chromosome E3	41,346,658	0	41,346,658
Chromosome F1	69,875,921	1	69,875,821
Chromosome F2	83,746,879	0	83,746,879
Chromosome X	126,453,223	19	126,451,323
Chromosome Y (SCR)	1,253,044	2	1,252,844
Chromosome Y Unlocalized (n=11)	2,222,635	0	2,222,635
Unplaced (n=21)	5,069,845	3	5,069,545

Supplementary Table 13. RepeatMasker repeat analysis summary.

**Total Sequence Masked**	**817,518,325 (bp)**		**33.67%**
**Elements**	**Number of Elements**	**Length Occupied (bp)**	**Percentage**
**SINEs**	**469,392**	**69,228,429**	**2.85%**
Alu/B1	-	-	0.00%
MIRs	461,742	68,320,782	2.81%
**LINEs**	**820,170**	**461,379,223**	**19.00%**
LINE1	455,565	364,763,156	15.02%
LINE2	310,104	84,495,495	3.48%
L3/CR1	40,779	8,846,541	0.36%
RTE	12,488	3,076,198	0.13%
**LRT elements**	**285,101**	**108,386,633**	**4.46%**
ERVL	86,921	39,232,030	1.62%
ERVL-MaLRs	145,860	50,918,315	2.10%
ERV_classI	28,392	12,440,852	0.51%
ERV_classIII	-	-	0.00%
**DNA elements**	**342,818**	**68,433,349**	**2.82%**
hAT-Charlie	193,901	36,348,164	1.50%
TcMar-Trigger	53,706	14,407,736	0.59%
Unclassified	3,729	587,155	0.02%
**Total interspersed Repeats**		**708,014,789**	**29.16%**
small RNA	141,131	10,704,033	0.44%
Satellites	2	462	0.00%
Simple Repeats	1,465,939	70,199,559	2.89%
Low complexity	531,854	28,455,522	1.17%

Supplementary Table 14. Summary of annotation liftover between the felCat9 reference and the Jungle cat assembly.

	**Protein Coding Genes**	
	**felCat9**	**Jungle Cat**
Chromosome A1	1,247	1,249
Chromosome A2	1,591	1,614
Chromosome A3	1,131	1,141
Chromosome B1	966	970
Chromosome B2	1,077	1,069
Chromosome B3	1,187	1,200
Chromosome B4	1,225	1,225
Chromosome C1	1,737	1,756
Chromosome C2	898	906
Chromosome D1	1,448	1,460
Chromosome D2	631	650
Chromosome D3	661	673
Chromosome D4	798	815
Chromosome E1	1,096	1,098
Chromosome E2	1,107	1,111
Chromosome E3	691	696
Chromosome F1	681	696
Chromosome F2	422	425
Chromosome X	781	792
Chromosome Y	21	13
ChrUn (n=32)	219	52
**Total**	**19,594**	**19,611**
**Jungle Cat Copies**	**203**	**1.04%**

Supplementary Table 15. Additional Jungle cat gene copies identified by Liftoff relative to felCat9.0 with Y chromosome single copy region annotations (Li et al, 2013).

Gene Name	Extra Copies	Gene Name	Extra Copies
A_RPS4Y	3	LOC101101494	1
ARV1	2	LOC102901082	1
B_RPS4Y	2	LOC102902070	1
C_RPS4Y	5	LOC105259672	1
CD8A	1	LOC105259679	1
d340_FLJ36031Y	3	LOC105259841	1
e268_FLJ36031Y	3	LOC105260280	1
GIMAP2	1	LOC105260290	2
GIMAP6	1	LOC105260348	2
GP1BB	1	LOC105260366	6
HSFX4	1	LOC105260391	1
HSFY	2	LOC105260530	1
IFNW4	1	LOC105260634	1
LOC101080402	1	LOC105260966	1
LOC101080615	1	LOC105260993	1
LOC101080874	1	LOC105261116	1
LOC101081393	1	LOC109493961	1
LOC101081959	1	LOC109493962	1
LOC101083228	1	LOC109496557	5
LOC101083281	2	LOC109496917	22
LOC101084708	1	LOC109496987	1
LOC101085613	1	LOC111556525	36
LOC101089503	2	LOC111556663	1
LOC101089637	1	LOC111556757	19
LOC101089971	1	LOC111556934	4
LOC101090249	1	LOC111557541	9
LOC101090443	1	LOC111558267	1
LOC101090556	1	LOC111558710	1
LOC101091647	1	LOC111558783	3
LOC101091653	1	LOC111560404	4
LOC101091766	1	LOC111561459	4
LOC101094994	1	LOC111561460	7
LOC101097517	1	LOC111561651	1
LOC101097790	1	LOC111561809	1
LOC101098529	1	MZT2B	1
LOC101099158	1	PNMA6A	1
LOC101100737	1	SEPT5	1
LOC101101043	1	SMPD4	1
LOC101101243	1	TMEM211	1

Supplementary Table 16. Assemblytics variant analysis comparing the Jungle cat assembly to the Fca-508 single haplotype domestic cat assembly.

Variant	Count	Total (bp)
**Insertion**		
50-500	18,943	3,647,523
500-10,000	1,016	2,708,148
Total	19,959	6,355,671
**Deletion**		
50-500	16,586	3,011,021
500-10,000	863	1,945,977
Total	17,449	4,956,998
**Tandem Expansion**		
50-500	1,647	336,364
500-10,000	271	446,894
Total	1,918	783,258
**Tandem Contraction**		
50-500	1,366	296,395
500-10,000	242	296,372
Total	1,608	592,767
**Repeat Expansion**		
50-500	11,526	3,390,482
500-10,000	8,371	15,139,307
Total	19,897	18,529,789
**Repeat Contraction**		
50-500	9,592	2,692,419
500-10,000	6,595	11,673,785
Total	16,187	14,366,204
**Total structural variants**		**77,018**
**Total affected bases**		**45.59 Mb**
**Total Gain**		**25.67 Mb**
**Total Loss**		**19.92 Mb**
**Difference**		**5.75 Mb**
